# Identification of T-cell exhaustion-related genes and prediction of their immunotherapeutic role in lung adenocarcinoma

**DOI:** 10.7150/jca.92839

**Published:** 2024-02-25

**Authors:** Chaoqun Lian, Feifan Li, Yiluo Xie, Linxiang Zhang, Huili Chen, Ziqiang Wang, Xinyu Pan, Xiaojing Wang, Jing Zhang

**Affiliations:** 1 Research Center of Clinical Laboratory Science, Bengbu Medical University, Bengbu 233030, China.; 2 Anhui Province Key Laboratory of Clinical and Preclinical Research in Respiratory Disease, Molecular Diagnosis Center, Department of Pulmonary and Critical Care Medicine, First Affiliated Hospital of Bengbu Medical University, Bengbu 233030, China.; 3 Department of Clinical Medicine, Bengbu Medical University, Bengbu 233030, China.; 4 Department of Genetics, School of Life Sciences, Bengbu Medical University, Bengbu 233030, China.; 5 Department of Medical Imaging, Bengbu Medical University, Bengbu 233030, China.; 6 Department of Tumor Radiotherapy, The First Affiliated Hospital of Bengbu Medical University, Bengbu 233030, China.

**Keywords:** Lung adenocarcinoma, T-cell exhaustion, Single-cell RNA-seq, Prognosis, Immunotherapy efficacy.

## Abstract

**Background:** Lung adenocarcinoma ranks as the second most widespread form of cancer globally, accompanied by a significant mortality rate. Several studies have shown that T cell exhaustion is associated with immunotherapy of tumours. Consequently, it is essential to comprehend the possible impact of T cell exhaustion on the tumor microenvironment. The purpose of this research was to create a TEX-based model that would use single-cell RNA-seq (scRNA-seq) and bulk-RNA sequencing to explore new possibilities for assessing the prognosis and immunotherapeutic response of LUAD patients.

**Methods:** RNA-seq data from LUAD patients was downloaded from the Cancer Genome Atlas (TCGA) database and the National Center for Biotechnology Information (GEO). 10X scRNA sequencing data, as reported by Bischoff P et al., was utilized for down-sampling clustering and subgroup identification using TSNE. TEX-associated genes were identified through gene set variance analysis (GSVA) and weighted gene correlation network analysis (WGCNA). We utilized LASSO-Cox analysis to establish predicted TEX features. External validation was conducted in GSE31210 and GSE30219 cohorts. Immunotherapeutic response was assessed in IMvigor210, GSE78220, GSE35640 and GSE100797 cohorts. Furthermore, we investigated differences in mutational profiles and immune microenvironment between various risk groups. We then screened TEXRS key regulatory genes using ROC diagnostic curves and KM survival curves. Finally, we verified the differential expression of key regulatory genes through RT-qPCR.

**Results:** Nine TEX genes were identified as highly predictive of LUAD prognosis and strongly correlated with disease outcome. Univariate and multivariate analysis revealed that patients in the low-risk group had significantly better overall survival rates compared with those in the high-risk group, highlighting the model's ability to independently predict LUAD prognosis. Our analysis revealed significant variation in the biological function, mutational landscape, and immune cell infiltration within the tumor microenvironment of both high-risk and low-risk groups. Additionally, immunotherapy was found to have a significant impact on both groups, indicating strong predictive efficacy of the model.

**Conclusions:** The TEX model showed good predictive performance and provided a new perspective for evaluating the efficacy of preimmunization, which provides a new strategy for the future treatment of lung adenocarcinoma.

## Introduction

Non-small cell lung cancer (NSCLC) accounts for 85% of all lung cancer cases, making it one of the most fatal cancers globally 1. Approximately 40% of all lung cancer cases are caused by lung adenocarcinoma (LUAD) 2. In the majority of cases, tumors are found to be locally advanced or metastatic disease. Although there have been significant advances in combination treatment strategies for LUAD, the average 5-year survival rate for LUAD is approximately 15% 3. In the last few years, immunotherapies that focus on immune checkpoints have been demonstrated to enhance the chances of survival in advanced non-small cell lung cancer (NSCLC), yet only a portion of patients exhibit a positive response to them 4. By recognizing potential prognostic biomarkers, it is possible to precisely categorize patients and determine those who are positively impacted by treatment. In recent years, there has been an increase in the number of studies using single-cell-seq and bulk-RNA-seq data to explore potential prognostic markers of LUAD, which has improved our understanding of tumorigenesis and progression. For example, Wang et al. identified arginine-substituted succinate-related genes 5; Song et al. developed an NK cell-based model to predict overall survival (OS) in patients with LUAD 6. However, LUAD has molecular heterogeneity and diverse tumor microenvironment (TME) compositions, making it difficult to fully reflect the heterogeneous TME and thus predict immunotherapy efficacy 7, 8. Therefore, it is necessary to develop predictive models and identify new biomarkers to predict prognosis and treatment efficacy.

A potential factor contributing to the low response rate in immunotherapy for lung adenocarcinoma could be T-cell exhaustion (TEX). An increasing number of studies indicate that the tumor microenvironment (TME) has a significant impact on the progression of cancer and the development of antitumor treatments, potentially contributing to resistance to immunotherapy 9. This is because exhaustion of CD8+ T cells (TEX), which leads to reduced function, is often associated with cancer immune escape 10. In cancer patients, T cells are constantly stimulated by prolonged exposure to persistent antigens and inflammation. Inactive T cells gradually lose their effector function and begin to lose the characteristics of memory T cells, a process known as T-cell exhaustion 11-13. T-cell exhaustion is considered a significant contributor to immune dysfunction in cancer patients. Several recent studies have indicated that inhibition of co-receptors on the surface of exhausted CD8+ T cells (CD8+Tex), including programmed death receptor 1 (PD-1), can lead to the rekindling of T cell cytolysis 14, 15. T cells have the potential to reactivate T cell cytolysis. Therefore, it is imperative that we focus on Tumour-Expressed X-linked proteins (TEX) and consider the reversal of TEX as a crucial factor in enhancing the objective remission rate of cancers during immunotherapy.

The objective of the study was to discover prognostic indicators of LUAD, which could predict the effectiveness of conventional therapies and suggest potential immunotherapies. TEX genes in LUAD were pinpointed through the use of single-cell RNA sequencing (scRNA-seq) datasets. A TEX prediction model (TEXRS) was developed in this example. Furthermore, the immunological features of the population defined by TEXRS are discussed. Finally, it has been determined that TEXRS is capable of effectively predicting the outcome and success of immunotherapy in LUAD patients. The analysis conducted has indicated that TEXRS showcases great potential as a prognostic model.

## Materials and methods

### Data collection and processing

Gene expression and clinical data were retrieved from TCGA (https://portal.gdc.cancer.gov/) and GEO databases (https://www.ncbi.nlm.nih.gov/). Initial normalization and log2 conversion of the original data were carried out with the Million Transcripts (TPM) method. This study utilised three distinct cohorts, with the TCGA-LUAD cohort employed as the training dataset and the GSE31210 (n=226) and GSE30219 (n=278) cohorts serving as validation datasets. The sample inclusion criteria for TCGA were 01A (Primary Tumor) type samples containing complete survival information. TEX-related genes were extracted from Zhang et al.'s article "Pan-cancer landscape of T-cell exhaustion heterogeneity within the tumor". The study uncovered a gradual breakdown of hierarchical function correlated with prognosis and treatment effectiveness within the tumor microenvironment 16 (Supplementary Table 1).

### Processing of single-cell data

The dataset for scRNA-seq regarding lung adenocarcinoma was obtained from the article "Single-cell RNA sequencing reverses distinct tumor microenvironmental patterns in lung adeno carcinoma"^17^. First, we used the "Seurat" R package to convert 10 × scRNA-seq data into Seurat objects and excluded cells of substandard quality and performed quality control (QC) by calculating the percentage of mitochondrial or ribosomal genes 18. We then identified highly variable genes for subsequent analysis. "Harmony" was used by us to remove batch effects. We constructed cell clusters using the "FindClusters" and "FindNeighbors" functions and visualized them using the "t-SNE" method. visualization using the "t-SNE" method. Finally, we performed cellular annotation based on marker genes for different cell types. the "AddModuleScore" function built into the Seurat package was used to quantify the activity of a specific set of genes in each cell. To analyze the differentially expressed genes (DEGs) between the two groups, we used the "FindMarkers" function in the Seurat package. Statistical significance of differentially expressed genes (DEGs) was calculated using the Wilcoxon test (p.adj < 0.05), and other parameters were set to default values. Genes differentially expressed between cells with high and low TEX scores at the single-cell transcriptome level were considered to be involved in TEX. these genes were subsequently included in the overall transcriptome level analysis of WGCNA. We also used the R package "CellChat"^19^ to perform cell interaction analysis.

### WGCNA identifies key modules

WGCNA (Weighted Correlation Network Analysis) is a systems biology approach for identifying patterns of genetic relationships between samples. WGCNA can be used to find highly synergistic genomes and to search for potential biomarker genes or therapeutic targets based on the endogenous nature of the genome and the linkage between the genome and the phenotype 20. WGCNA can be used to search for highly synergistic genomes and to find potential biomarker genes or therapeutic targets based on their endogenous nature and the association between genome and phenotype.

### Risk prediction model construction and validation

LASSO regression analysis of training set data was performed using the R package "glmnet to obtain the best results. Multiple regression Cox analysis was performed on 9 TEX-related genes. We then calculated each patient's risk score. The formula was as follows: Risk score = 0.107*CCL20 + -0.087 * GDF15 + -0.063 * BTG2 + -0.069 * METTL7A + -0.144 * PERP+ -0.229 * CTLA4+ 0.22 * KRT18 + 0.162 * KYNU + -0.059 * PRKCH. Kaplan-Meier survival analyses were carried out and the working characteristic curve of the subject were established. To test the predictive power of the model, we evaluated its prognosis, sensitivity and specificity in the experimental group. We then validated it in the GSE31210 and GSE30219 cohorts based on a risk score formula.

### Independent prognostic analysis and column chart construction

Univariate and multivariate Cox regression analyses were conducted to test if TEX characteristics can function as independent predictors among patients with LUAD. The "rms" R package was used to generate column charts, predicting 1-, 3-, and 5-year OS among clinical patients according to age, grade, gender, stage, T-stage, and risk score. The calibration study verified the precision of the column chart predictions.

### Single-sample genome enrichment analysis (ssGSEA) and genome enrichment analysis (GSEA)

ssGSEA is a widely used method to quantify the enrichment score for a specific set of genes in a single sample. The ssGSEA score for each sample reflects the extent to which a particular gene set is systematically up- or down-regulated in the sample. In this study, GSVA was used 21 ssGSEA in this study to obtain a TEX score for each TCGA-LUAD sample. The HALLMARK and KEGG pathways were also analyzed using the "ClusterProfiler" R package 22. Using the "c2.cp.kegg.v7.4.symbols.gmt" and "h.all.v2023.2.Hs.symbols" gene sets in MSigDB, the GSVA and GSEA algorithms were used to "c2.cp.kegg.v7.4.symbols.gmt" and "h.all.v2023.2. Hs.symbols" were analysed for differences in enrichment pathways between risk groups.

### Analysis of genomic variation among TEXRS risk subgroups

Mutation with built-in tumor heterogeneity (MATH) is a method to quantify intra-tumor heterogeneity (ITH) based on the distribution of mutant alleles. The prognostic significance of MATH has been investigated in a wide variety of tumors, including head and neck, colorectal, and breast cancers 23-26. In this investigation, the MATH score was computed for every LUAD patient using the previously specified method, and survival analysis was carried out according to their MATH scores. We generated waterfall plots to display mutations in both high- and low-risk groups of LUAD patients with TEXRS by leveraging the R package "maftools". Additionally, we computed the TMB score for each patient diagnosed with LUAD and examined the link between TMB, survival analysis, and high and low-risk groups.

### Correlation analysis of the TEX model with the immune microenvironment

To estimate the immunity score, stroma score and 22 different types of immune infiltrating cells, the R packages "ESTIMATE" and "CIBERSORT" were used 27, 28. Single sample immune cell infiltration scores were also quantified using single sample gene set enrichment analysis (ssGSEA) based on the R package GSVA. Finally we compared the mRNA expression levels of immune checkpoint inhibitory molecules. TIDE was used to predict tumor immunotherapy effects 29, TIDE score data were obtained from the TIDE website (http://tide.dfci.harvard.edu/).

### Immunotherapy prediction and chemosensitivity analysis

We collected three GEO immunotherapy cohorts (GSE78220^30^ GSE35640^31^ and GSE100797^32^), along with the IMvigor210 cohort, to investigate the relationship between TEX characteristics and immunotherapy. Data processing was carried out using the "IMvigor210CoreBiologies" R package from the IMvigor210 cohort 33. We used the "IMvigor210CoreBiologies" R package from the IMvigor210 cohort to process data. Furthermore, to establish the immunogenicity based on immunomodulators, immunosuppressive cells, MHC molecules, and effector cells, we utilised the Immunophenoscore (IPS) algorithm. This algorithm calculates the IPS score using the impartial gene expression of a representative cell type through a machine-learning methodology. A higher IPS score indicates an improved response to immunotherapy. IPS scores for patient samples of TCGA-LUAD were acquired from The Cancer Immunome Atlas (TCIA) database (https://tcia.at/home).

### Cell line culture and qRT-PCR

All cells were cultured at 37°C in an incubator with 5% CO2 atmosphere. Normal human lung cell line 2B, lung adenocarcinoma cells H1299 and A549 were obtained from the Chinese Academy of Sciences (Shanghai, China). Cell culture media, plates and dishes were from Thermo Fisher Scientific (Invitrogen, USA) and Corning Inc. 2B cells, H1299 cells and A549 cells were detached and inoculated into 60 mm dishes overnight at an initial density of 1 × 106 cells/well. Subsequently, SYBR Green qPCR mix (Vazyme, China) was used to synthesize cDNA for real-time PCR. Our results were analyzed using the comparative Ct method and the Ct values of each gene were normalized by the Ct reads of the corresponding GAPDH. All data are expressed as mean ± standard deviation (SD) of three independent experiments, and primer sequences are shown (Supplementary Table 2).

### Statistical analysis

All statistical analyses were performed using R software (version 4.0.2). Wilcoxon test was used to compare the differences between groups. The log-rank test was used to compare Kaplan-Meier survival curves. Univariate and multivariate Cox analyses were performed to establish independent prognostic factors. All P values were two-sided and less than 0.05% were considered statistically significant. All P values were two-sided and less than 0.05 were considered statistically significant.

## Results

### Identification of TEX-related genes from single-cell transcriptomes

**Figure 1:**
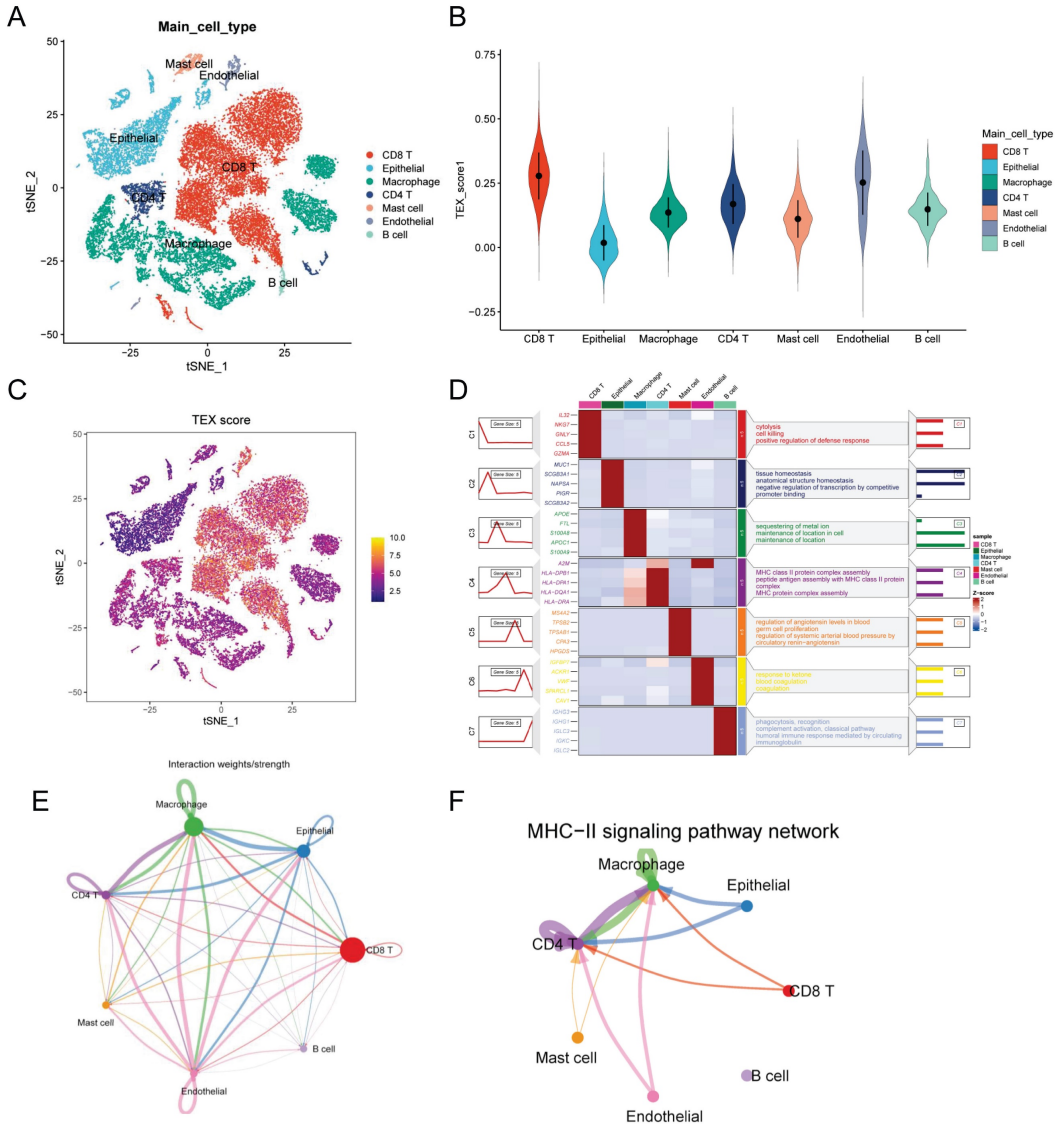
Identification of TEX-related genes from single-cell transcriptomes. **(A)**: t-SNE plot showing cell types recognized by marker genes. **(B)**: T-cell exhaustion activity score (TEX) for each cell. **(C)**: Distribution of TEX scores in different cell types. **(D)**: Heatmap showing the 5 most important marker genes in each cell cluster. **(E)**: Cell communication network. **(F)**: MHC-II communication between each cell.

After filtering data from Philip Bischoff et al, we used six single-cell datasets and 27,066 cells. A total of 18 distinct cell subsets were identified by TSNE analysis (Supplementary Figure 1A). We annotated these 18 cell subpopulations and identified 7 cell types, including macrophages, epithelial cells, endothelial cells, CD4 T cells, CD8 T cells, mast cells, and B cells (Figure 1A). To quantify the activity of T cell failure in different cell types, we used the AddModeleScore function in the Seurat software package to calculate the expression levels of T cell failure-associated gene sets in all cells (Figure 1C). Among the seven cell types, we observed a significant increase in TEX activity in endothelial cells, CD8 T cells and CD4T cells (Figure 1B). Based on the TEX activity, we divided the cells into high TEX and high and low TEX groups and identified two groups of 2063 differentially-expressed genes (DEGs) for further analysis (Supplementary Table 3). The heatmap show the top four marker genes for each cell population (Figure 1D). We investigated cellular communication networks by calculating communication probabilities (Figure 1E, Supplementary Figure 1B). In addition, we inferred that cell-to-cell communication networks are based on specific pathways and ligand receptors. We found that the MHC-II signaling pathway plays a critical role in CD4T cell communication networks (Figure 1F). The immune system largely recognizes tumor cells complexity through the major histocompatibility complex (MHC). High expression of MHC-II in tumors is key to T lymphocyte antigen presentation, and the role of CD4+ T lymphocytes in antitumor immunity is gaining traction 18.

### Identification of TEX-related genes in the bulk-RNA-seq transcriptome

**Figure 2:**
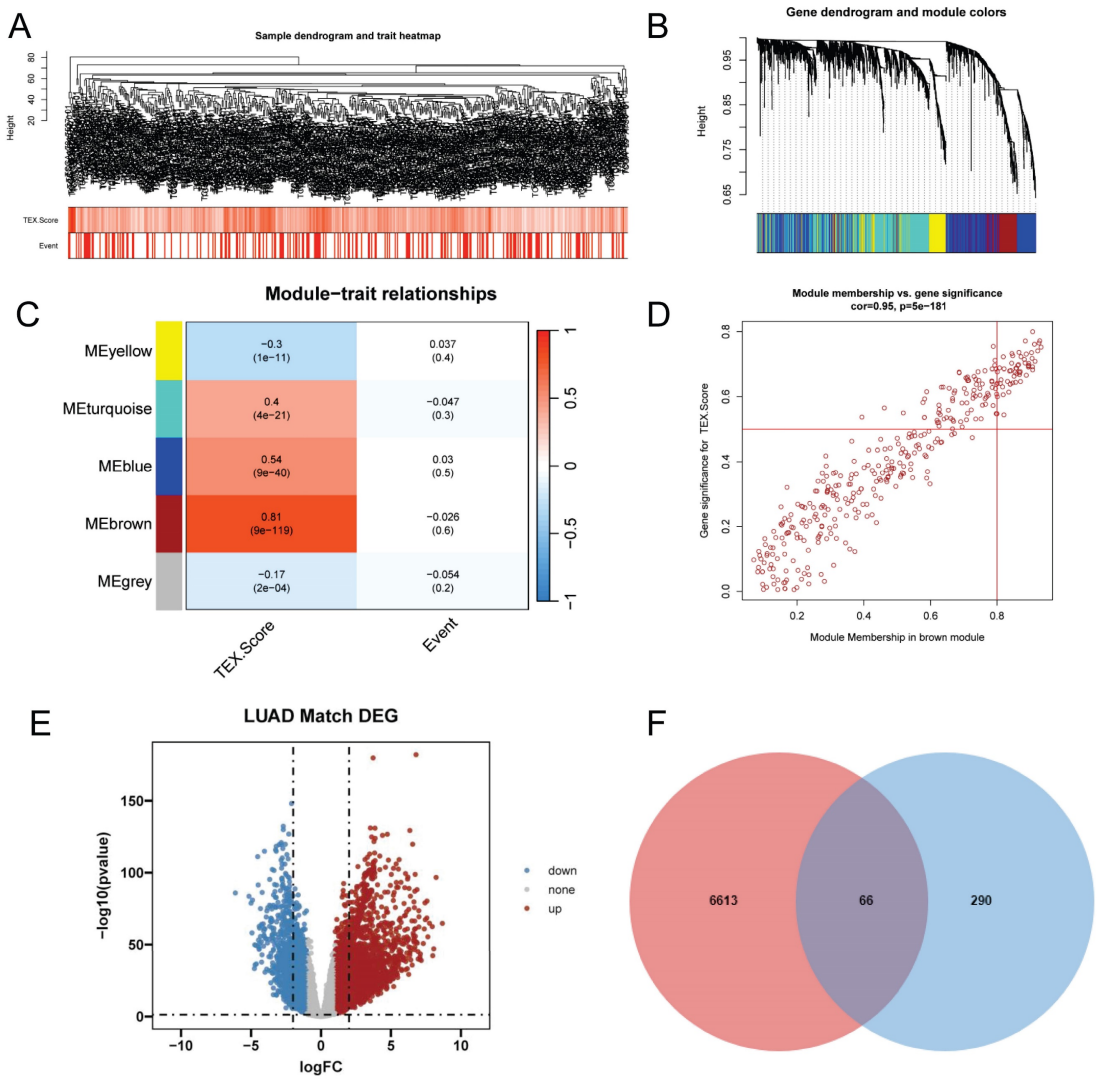
Identification of TEX-related genes from the bulk transcriptome. **(A)** Dendrogram showing hierarchical clustering of TCGA-LUAD samples, with the heatmap at the bottom indicating the TEX score for each sample, as calculated by the ssGSEA algorithm. **(B)** Clustering dendrogram analysis of WGCNA. **(C)** Heatmap of module features showing that MEbrown modules are closely associated with TEX features. **(D)** Scatter plot showing the relationship between gene significance (GS) and module membership (MM) in brown modules. **(E)** Volcano plot showing the results of difference analysis between TCGA-LUAD tumor samples and normal samples. **(F)** Venn diagram showing the crossover genes between MEbrown modules and DEGs in bulk-RNA-seq.

The ssGSEA analysis was used to obtain TEX activity scores for each TCGA-LUAD sample, which served as the phenotypic data for the subsequent WGCNA analysis. Modules significantly correlated with TEX scores were identified by performing WGCNA analysis on the TCGA-LUAD dataset. Following the elimination of outlier samples, a co-expression network was developed utilising the 2063 DEGs identified at the single-cell-seq level (Figure 2A). To guarantee that the topological network conformed to the scale-free principle, we selected the optimal soft threshold for power = 4 (Supplementary Figure 1C). We acquired 5 modules by defining the minimum module gene count as 60 and medissres as 0.25 (Figure 2B). Our findings reveal that the MEbrown modules were significantly correlated with TEX scores in bulk-RNA-seq (cor = 0.81, Figure 2C). Furthermore, a correlation analysis was conducted on gene significance (GS) and module membership (MM) for the brown module, revealing a significant correlation (cor = 0.95, p = 5e-181, Figure 2D). This implies that the brown module's genes might have functional significance related to T-cell exhaustion. Additionally, the volcano plot (Figure 2E) illustrates the differentially expressed genes (DEGs) between tumor and normal lung tissues in TCGA-LUAD bulk-RNA-seq dataset (|logFC|> 1 and p.d adj < 0.05). We identified and selected 66 genes, which we named T-cell exhaustion-related genes (TEXRgenes), from the 356 genes in the brown module that were analyzed in combination with DEGs from bulk RNA sequencing (Figure 2F). Our analysis suggests that these TEXRgenes are involved in T-cell exhaustion (TEX) at both bulk-RNA-seq and single-cell transcriptome levels. This was also confirmed by GO enrichment analysis of TEXRgenes (Supplementary Figure 1D).

### TEX modeling and external validation

**Figure 3:**
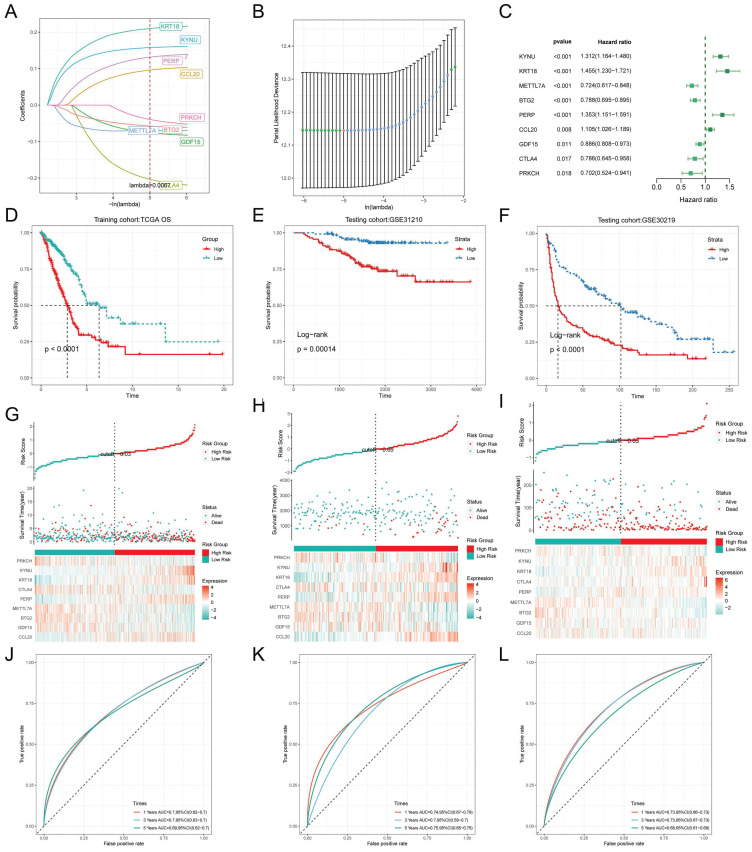
TEX modeling and prediction. **(A)** Lasso regression curves for TEX to avoid overfitting. **(B)** 10-fold cross-validation of variable selection using Lasso. **(C)** Forest plot showing univariate Cox results. (D-F) KM curves comparing overall LUAD patients between the LR and HR groups in the TCGA-LUAD **(D)** cohort, GSE31210 **(E)** and GSE30219 **(F)** cohorts. (G-I) Distribution of risk scores and patient survival between the LR and HR groups in the TCGA-LUAD **(G)** cohort, GSE31210 **(H)** and GSE30219 **(I)** cohorts. (J- L) Time-dependent ROC curve analysis in the TCGA-LUAD **(J)** cohort, GSE31210 **(K)** and GSE30219 **(L)** cohorts.

To exclude co-expressed TEX genes and avoid overfitting, we constructed a predictive prognostic model consisting of TEX genes by lasso regression analysis. They were CCL20, CTLA4, KYNU, KRT18, PRKCH, PERP, BTG2, METTL7A, and GDF15 (Figure 3A, B). A linear prediction model was developed based on the weighted regression coefficients of the nine prognostically relevant TEXs, which were calculated as follows: risk score = (0.107*CCL20+-0.087*GDF15+-0.063*BTG2+0.069*METTL7A+0.144*PERP+0.229*CTLA4+0.22*KRT18+0.162*KYNU+-0.059*PRKCH). To demonstrate the stability and reliable generalization of our model, the TCGA-LUAD cohort was used as the internal training set, and the GSE31210 and GSE30219 cohorts were used as the external validation cohorts. Based on the same risk formula, risk scores were calculated for each sample in the TCGA training cohort and the GEO validation cohort, respectively, and we could find that when the risk of patients with LUAD was elevated in both cohorts, the patients exhibited a survival disadvantage of reduced OS and increased mortality (Figure 3G-I). Based on the median risk score, we could divide the patients into two subgroups, HR (High Risk) and LR (Low Risk), to explore the prognostic differences between the HR and LR groups. The Kaplan-Meier curves showed significant prognostic differences between HR and LR patients in each of the two cohorts, with a more pronounced survival advantage for the patients in the LR group (Figure 3D-F). The ROC curves were used as a predictor of the patient's survival at 1, 3, and 5 year time, with AUCs of 0.7, 0.7, and 0.69 for the TCGA-LUAD cohort, respectively. The AUCs of 0.74, 0.7, and 0.75 for the GSE31210 cohort and 0.73, 0.73, and 0.66 for the GSE30219 cohort (Figure 3J-L) indicated that the model had a good predictive effect. In addition, we obtained clinical information for the HR and LRgroups (Table 1).

### Creation of column line diagrams based on TEX model combined with clinical features

**Figure 4:**
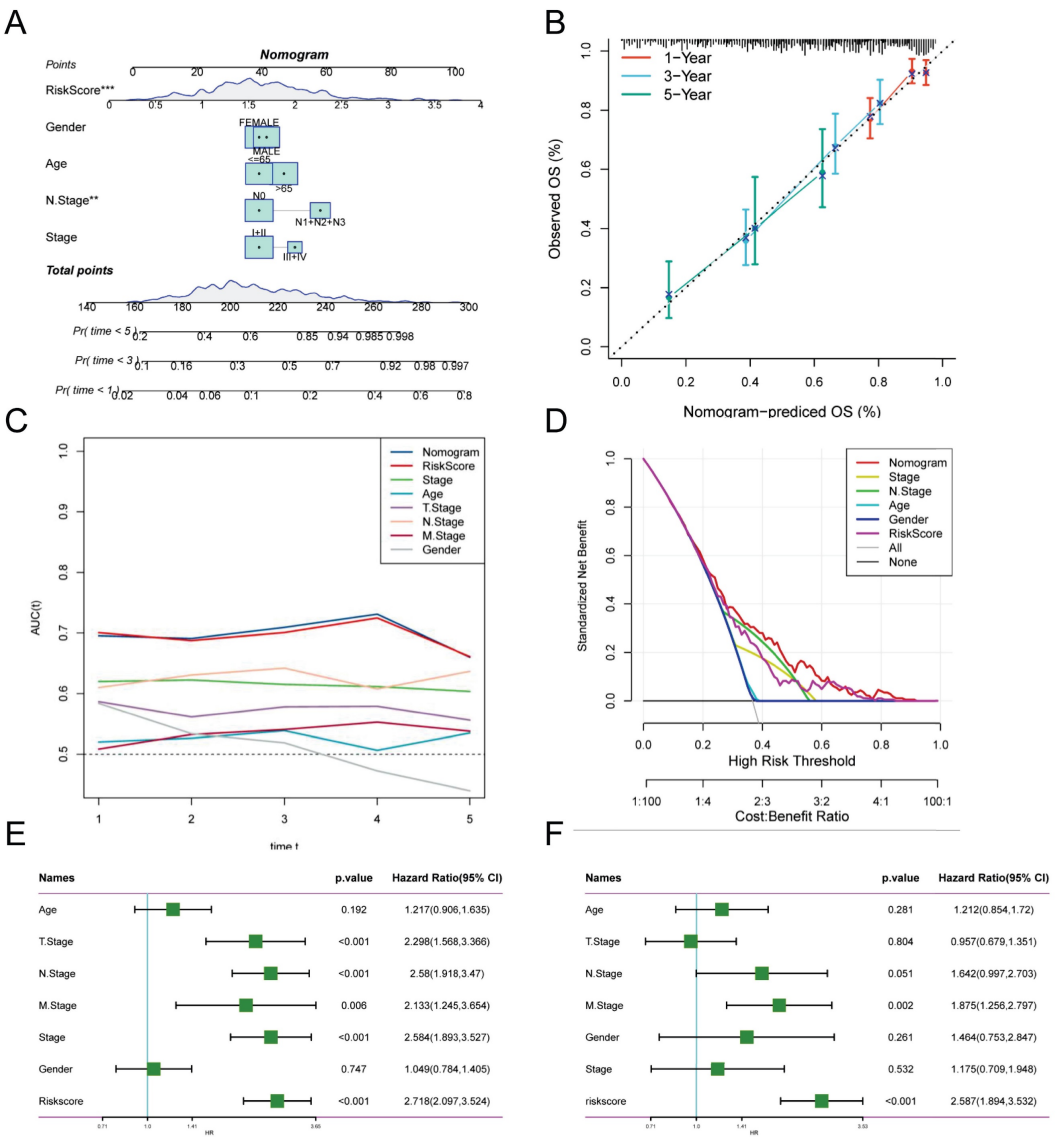
Creation of column-line plots based on the TEX model combined with clinical characteristics. **(A)** Column plots combining age, grade, sex, N-stage, total stage, and risk score. **(B)** Calibration curves for the constructed 1-, 3-, and 5-year survival column plots. **(C)** Time-dependent ROC curve analysis. **(D)** DCA decision curve analysis. **(E)** Univariate and **(F)** multivariate COX regression analysis of characteristics and different clinical features.

Risk scores and clinical metrics were combined to develop column-line plots as predictors of 1-, 3-, and 5-year prognostic survival probabilities (Figure 4A). Subsequently, calibration curve analysis displayed patients' 1-, 3-, and 5-year OS prediction curves to be closely similar to the ideal 45-degree calibration line, thus indicating the column charts' stability to be excellent (Figure 4B). The TCGA cohort was subjected to a TimeROC analysis, which revealed that the AUCs of the column charts and risk scores surpassed the other metrics (Figure 4C). Nomogram and risk score showed better predictive efficacy compared to other clinical characteristics, as demonstrated by the Decision Curve Analysis (DCA) (Figure 4D). To assess the reliability and clinical value of biometric features constructed using TEX as prognostic predictors, we compared the risk scores of every LUAD patient with two standard clinical measures. Subsequently, we observed the correlation between each factor and the patient's prognosis through consecutive univariate and prediction analyses, employing multivariate Cox analysis. Upon analysing the results, it becomes apparent that staging, T-staging and risk score (P < 0.001) are all prognostic factors that have a statistically significant association with patient prognosis in the univariate cox analysis (Figure 4E). Nevertheless, following multivariate cox analysis, only the risk score (P < 0.001) retains significant association (Figure 4F). These findings indicate that our TEX model is a more effective and impactful clinical decision-making tool, better suited to predicting the prognosis of LUAD patients in clinical settings.

### Clinical Relevance and Survival Analysis of TEX in LUAD Patients

**Figure 5:**
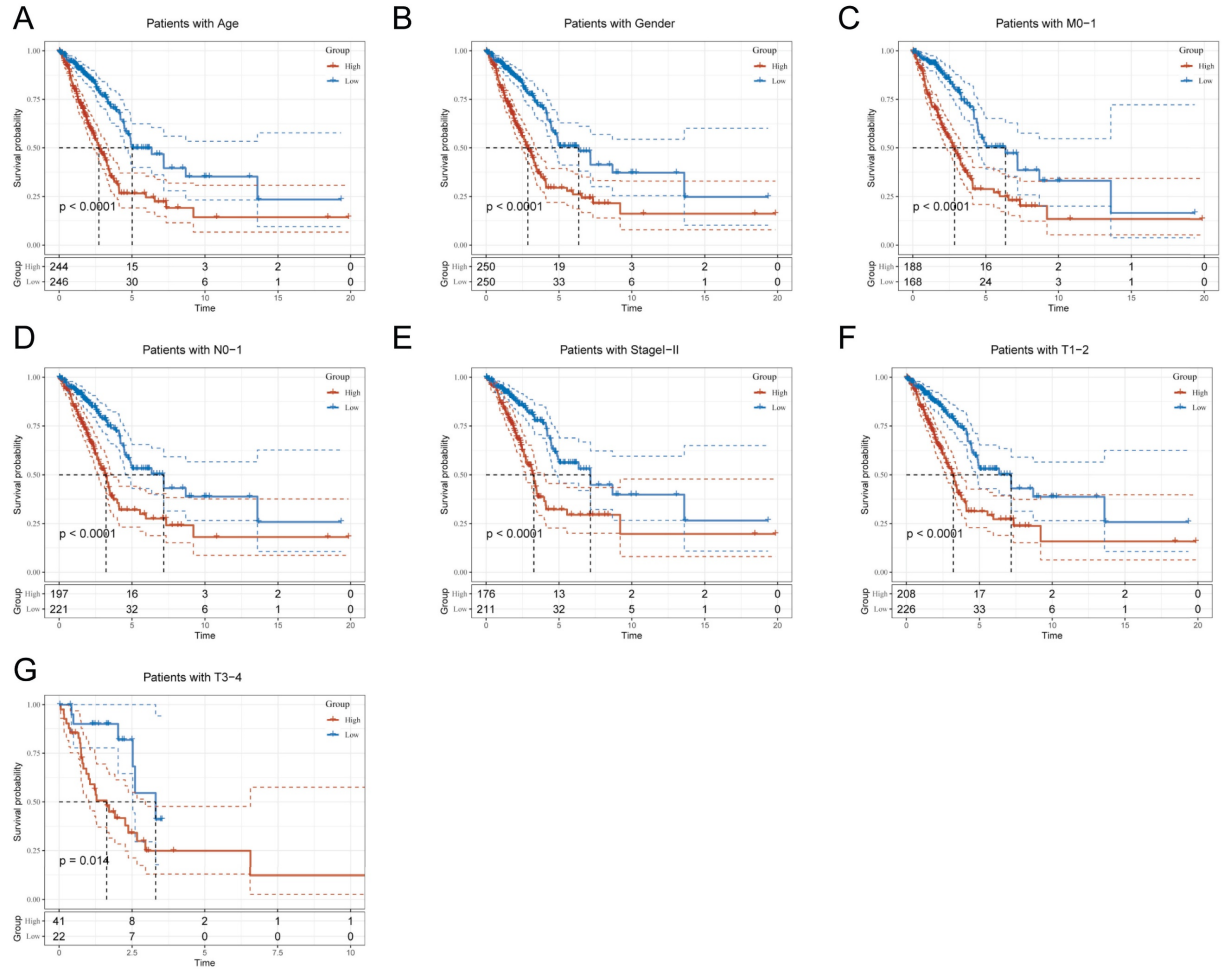
Clinical relevance and survival analysis of TEX in LUAD patients. **(A)** Age. **(B)** Gender. **(C)** Pathologic M-staging. **(D)** N-staging. **(E)** Total staging (I-II). **(F)** T-staging (T1-2). **(G)** T-staging (T3-4).

Due to the notable divergence in individual clinical characteristics of overall survival (OS) between the high risk (HR) and low risk (LR) groups, we have classified LUAD patients into five distinct subgroups based on clinical traits. These subgroups comprise age, pathological stage (I-II and III-IV), gender (female and male), pathological M-stage (M0-1), N-stage (N0-N1), and T-stage (T1-2 and T3-4). This categorization aims to attain a more accurate and specific analysis of potential disparities and similarities between these groups. It is worth noting that LR patients exhibited a notable advantage in terms of longer survival time compared to HR patients in all subgroups (as shown in Figure 5A-G and Supplementary Figure 2A-H). Our analysis of the results has led us to conclude that the TEX model is a dependable clinical prediction tool.

### Gene set enrichment analysis

**Figure 6:**
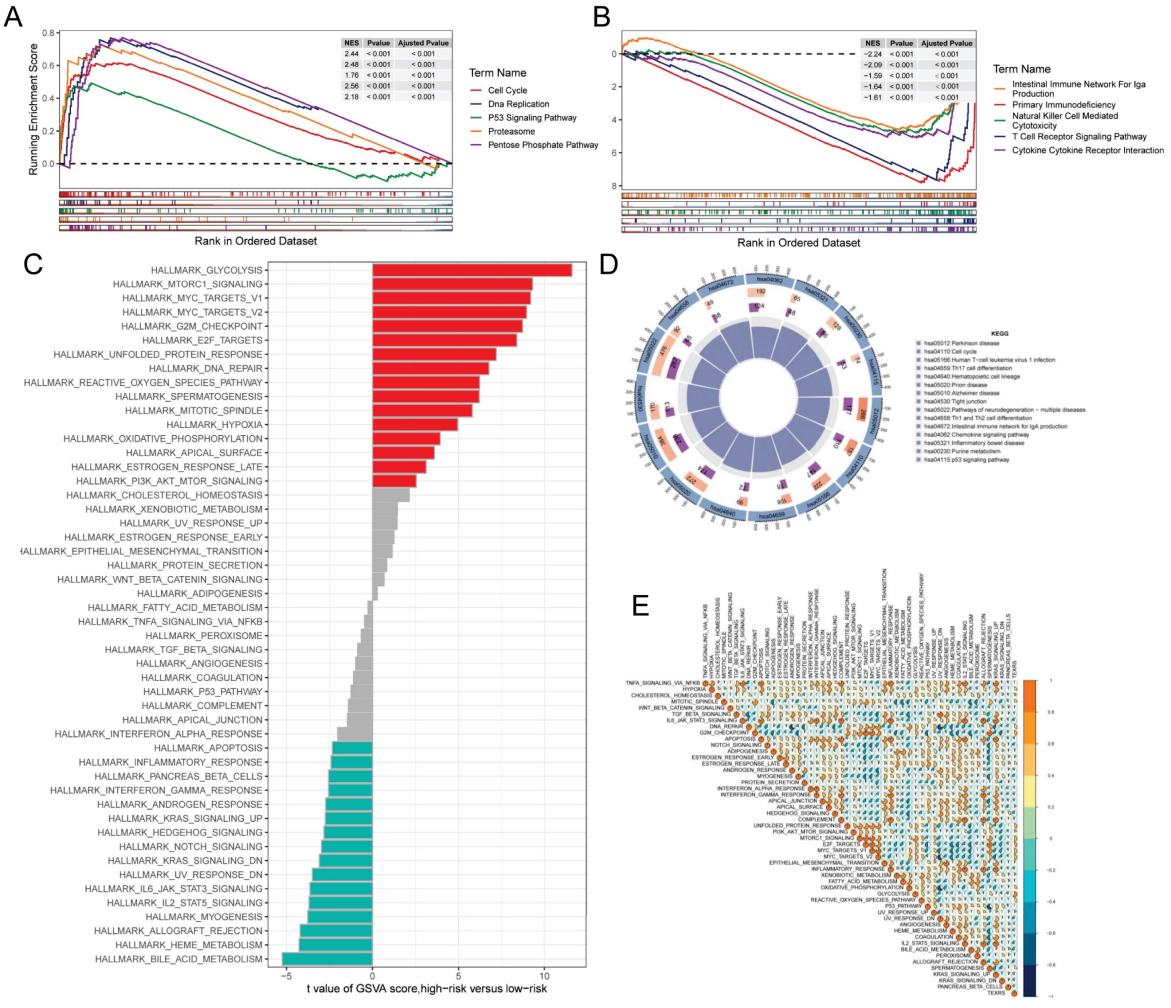
Gene set enrichment analysis. **(A)** KEGG gene set enriched in the high TEXRS group. **(B)** KEGG gene set enriched in the low TEXRS group. **(C)** Differences in HALLMARK pathway activity between high and low risk groups for GSVA scores. **(D)** Circle plot demonstrating differential gene enrichment of the KEGG pathway between the two groups. **(E)** Correlation between risk score and marker pathway activity of GSAV score.

GSEA was used to identify KEGG gene sets enriched in both TEXRS groups. The GSEA plot shows only the first 5 routes. The gene set of the low TEXRS group was enriched for immune-related pathways such as T Cell Receptor Signaling Pathway, Intestinual Immune Network For IGA Production, etc. whereas the gene set of the high TEXRS group was enriched for cell cycle- and cancer-related pathways (Figure 6A, B). GSVA analyzed the differentially enriched HALLMARK pathways between the two groups (Figure 6C). The results showed that the high-risk group was predominantly enriched to oncogenic pathways, whereas the low-risk group was predominantly enriched to immune-related pathways. Differential analysis of the high- and low-risk groups showed that the differential genes were mainly enriched to the cell cycle, p53 signaling pathway, etc. (Figure 6D). These findings were further supported by correlation analysis between TEXRS and hallmarks pathway scores (Figure 6E), suggesting that TEXRS is closely associated with cancer-related biological processes and metabolic pathways.

### Mutations in genes associated with TEXRS between low and high-risk groups

**Figure 7:**
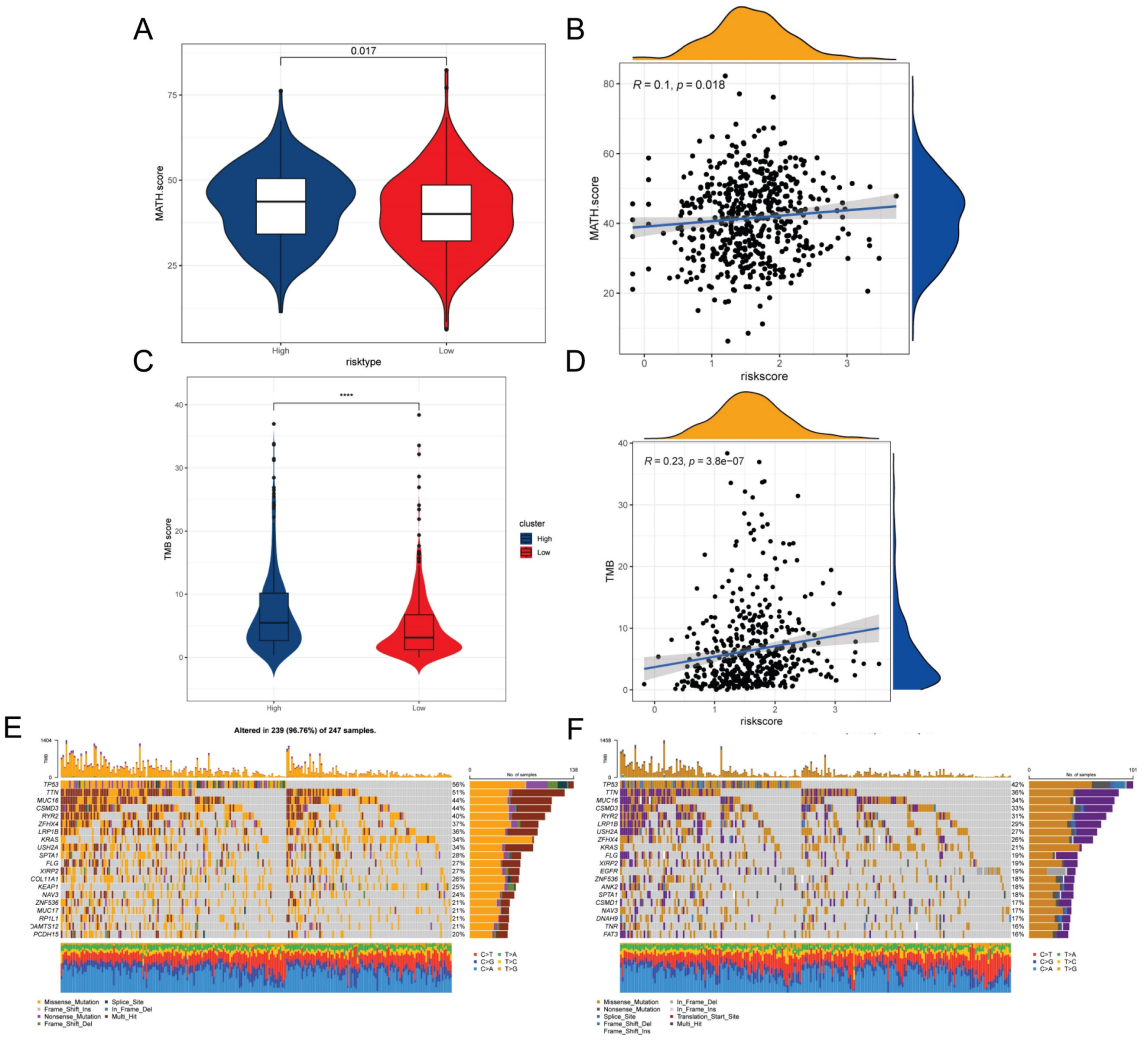
Mutations associated with TEXRS between low and high-risk groups. **(A)** Violin plot showing the difference in mutant allele tumor heterogeneity (MATH) scores between the high- and low-risk groups. **(B)** Correlation between risk score and MATH.Score. **(C)** Violin plot demonstrating the difference in TMB between high and low risk groups. **(D)** Correlation between TMB and risk score. **(E)** Mutation analysis of the high-risk group. **(F)** Mutation analysis of the low-risk group.

Intra-tumor heterogeneity (ITH) is a well-known genomic feature of cancer caused by mutation 34 accumulation resulting in cancer. ITH has been shown to be associated with malignancy and increased resistance to chemotherapy 35. In this study, we measured ITH in LUAD patients using the Mutant Allele Tumor Heterogeneity (MATH) algorithm; higher MATH scores were associated with higher ITH. The MATH score was higher in the high-risk group of LUAD patients (Figure 7A). We further explored the relationship between ITH and risk scores of LUAD patients, Spearman correlation analysis was performed in this study, and a significant positive correlation was found between risk scores and MATHscore, suggesting that the combination of these two metrics can better assess the prognosis of LUAD patients (Figure 7B). In addition, the TMB analysis of the HR and LR groups showed a significant difference between the two, with a higher TMB in the HR group (Figure 7C). There was also a significant positive correlation between risk score and TMB (Figure 7D). It is well known that genetic mutation is a condition for tumorigenesis. In the TCGA database, we visualized and correlated the somatic mutation data based on TEX signatures combined with HR and LR groups. The three genes with the highest mutation frequencies in the HR group were TP53 (56%), TTN (51%), and MUC16 (44%) (Figure 7E, F).

### TEX risk score predicts tumor microenvironment and immune cell infiltration

**Figure 8:**
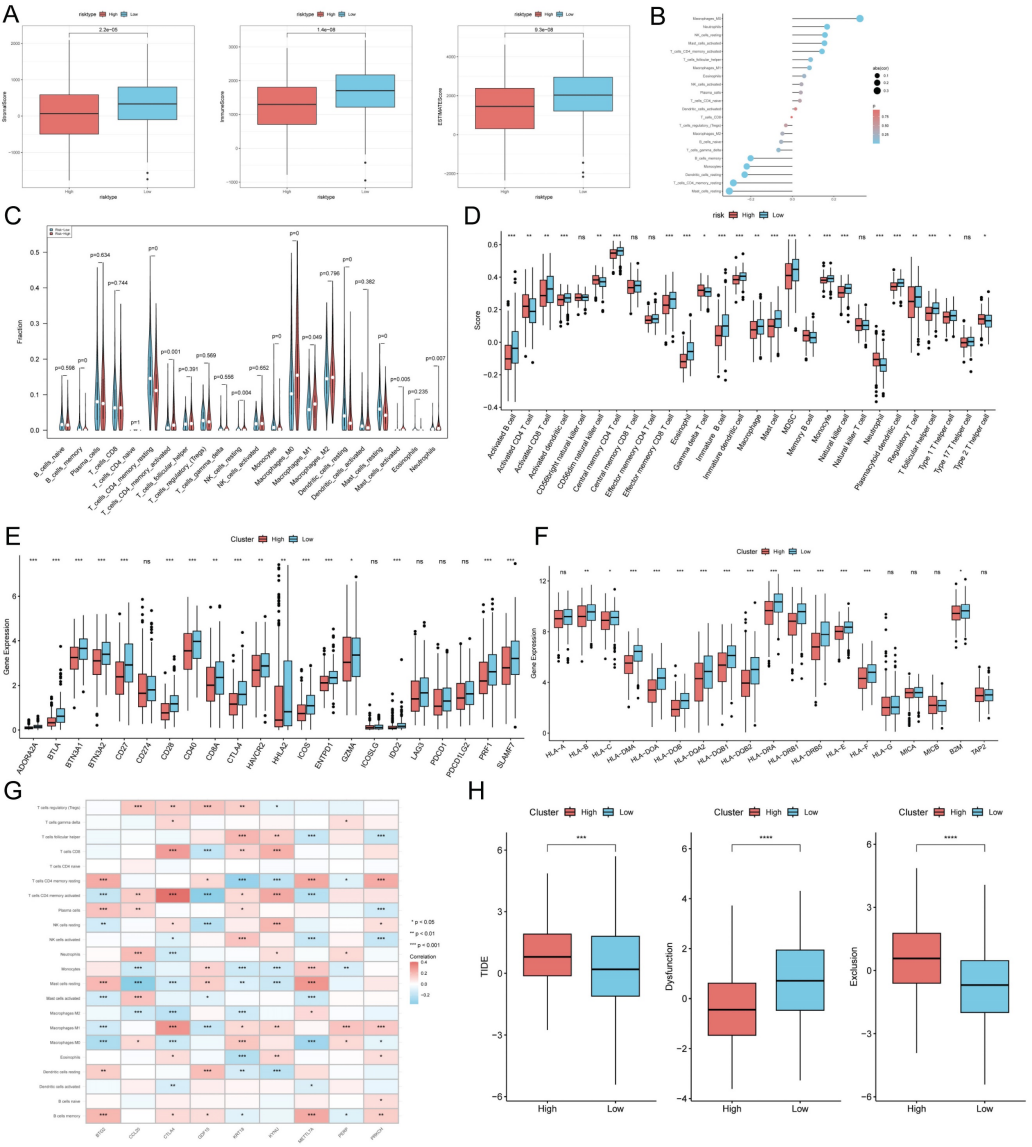
TEX risk score predicts tumor microenvironment and immune cell infiltration. **(A)** Immunity score, ESTIMATE score and stroma score were used to quantify the different immune status between high and low risk groups. **(B)** Correlation analysis of TME infiltrating cells with TEXRS. **(C)** Abundance of each TME-infiltrating cell type was quantified by the CIBESORT algorithm and the ssGSEA algorithm **(D)** between high and low risk groups. **(E)** Differential expression of various immune checkpoints in high and low risk groups. **(F)** Differential expression of HLA molecules in high and low risk groups. **(G)** Relationship between TME-infiltrating cells and TEXRS genes. **(H)** TIDE assessment of immunotherapy escape in high- and low-risk groups.

It has been established that interactions between cancer cells and TME are critical for tumor progression and dissemination 36. Therefore in this study, to assess the immune infiltration status of LUAD samples, we used the ESTIMATE algorithm to calculate the immunity score, stromal score and ESTIMATE score for the TEXRS risk subgroup. The immunity score stromal score and ESTIMATE score were significantly higher in the low-risk group (Figure 8A). Next, we used the CIBERSORT results to screen for immune cell types significantly associated with TEXRS by Spearman's correlation analysis (Figure 8B). To further analyze the difference in specific immune cell infiltration between the high-risk and low-risk groups, we quantified the abundance of immune cell infiltration in each sample using the CIBERSORT algorithm, which showed a larger proportion of T cells and macrophages (Figure 8C). Similar results were obtained by applying the ssGSEA algorithm for validation (Figure 8D). Previous studies have reported that high expression of immune checkpoints is associated with better response to immune checkpoint inhibitor (ICI) therapy 37-39. Therefore, we analyzed the differences in immune checkpoints on the basis of risk scores, and found that the expression was higher in the low-risk group (Figure 8E) 40 and we also compared the molecular differences of HLA between different groups (Figure 8F). In addition, we found that nine genes within TEXRS were highly correlated with tumor-infiltrating immune cells, among which CTLA4 and KYNU were positively correlated with T-cell CD8, and METTL7A was positively correlated with M2 macrophages (Figure 8G) The TIDE results demonstrated that the high-risk group had higher scores, which may imply that the high-risk group may have stronger immune escape (Figure 8H).

### Predicting and validating the efficacy of immunotherapy

**Figure 9:**
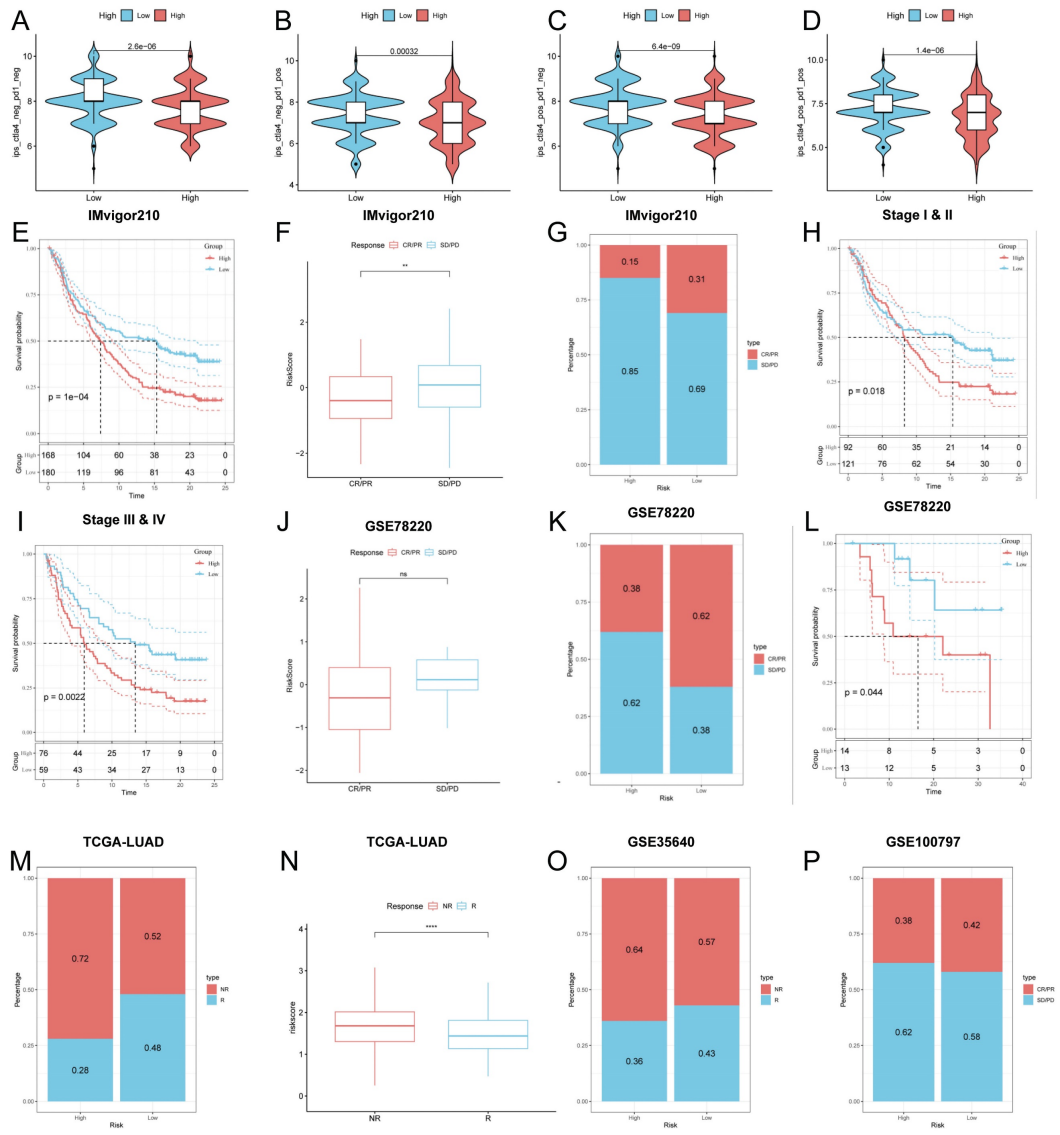
Predicting and validating the efficacy of immunotherapy. **(A-D)** IPS scores in the high- and low-risk groups **(A)** ips_ctla4_neg_pd1_neg **(B)** ips_ctla4_neg_pd1_pos **(C)** ips_ctla4_pos_pd1_neg **(D)** ips_ctla4_pos_pd1_pos. **(E)** Survival curves for the IMvigor 210 cohort in the HR group and LR group survival curves. **(F)** Box line plot depicting the difference in risk scores between CR/PR patients and SD/PD patients in the IMvigor210 cohort. **(G)** Proportion of CR/PR or SD/PD patients receiving immunotherapy in the high and low risk groups of the IMvigor210 cohort. (H, I) km curves for the high and low risk groups of the IMvigor210 staging. **(H)** Stage I-II **(I)** Stage III-IV. **(J)** Box line plot depicting the difference in risk scores between CR/PR patients and SD/PD patients in the GSE78220 cohort. **(K)** Proportion of CR/PR or SD/PD patients receiving immunotherapy in the high and low risk groups of the GSE78220 cohort. **(L)** Survival curves for HR and LR in the GSE78220 cohort. **(M)** Proportion of patients with R or NR who received immunotherapy in the high and low risk groups of the TCGA-LUAD cohort. **(N)** Box line plot depicting the difference in risk scores between R patients and NR patients in the TCGA-LUAD cohort. **(O)** Proportion of R or NR patients receiving immunotherapy in the high and low risk groups of the GSE35640 cohort. **(P)** Proportion of CR/PR or SD/PD patients receiving immunotherapy in the high and low risk groups of the GSE100797 cohort.

To further validate our results, we analyzed the IPS scores obtained from the TCIA database. Higher IPS scores predicted a better response to ICI therapy, including PD-1 inhibitor and CTLA4 inhibitor therapy, and were categorized into four categories: ips_ctla4_pos_pd1_pos, ips_ctla4_pos_pd1_neg, ips_ctla4_neg_pd1_pos and ips_ctla4_neg_pd1_neg. Our results showed that all four categories were significantly elevated in the low-risk group, suggesting that patients in the low-risk group responded better than patients in the high-risk group to anti-CTLA4 therapy as well as to the combination of anti-pd -1 and anti-CTLA4 therapy (Figure 9A-D). In addition to test the potential of risk scores in predicting immunotherapy in a real immunotherapy cohort, we selected four groups of patients receiving immunotherapy (IMvigor210, GSE78220, GSE35640, and GSE100797), and the results showed that the patients in the low-risk group with complete remission/partial remission (complete response/partial response, CR/PR) ratio was significantly higher and the number of responders to immunotherapy was also higher in the low-risk group than in the high-risk group (Figure 9F, G, J, K, M, N, O, P). Similarly, in all four cohorts, patients at lower risk may have a better prognosis (Figure 9E, H, I, L). All these results imply that the low-risk group has a favorable immunotherapy effect.

### Identification of key regulatory genes in the TEX model

**Figure 10:**
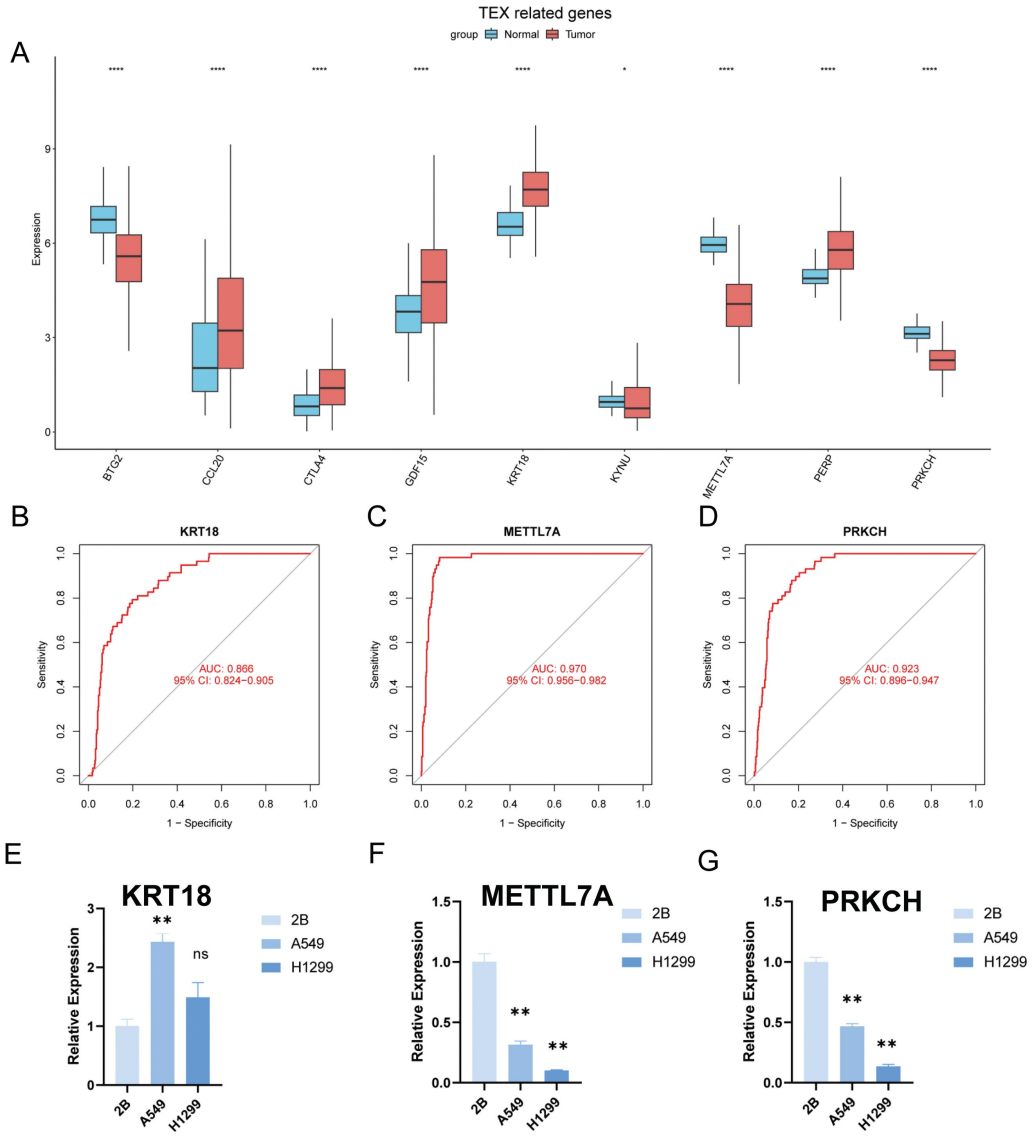
Identification of key regulatory genes in the TEX model. **(A)** Box-and-line plot demonstrating the expression of TEXRS genes in cancer and paracancer. **(B)** ROC diagnostic curve of KRT18. **(C)** ROC diagnostic curve of METTL7A. **(D)** ROC diagnostic curve of PRKCH. (E-G) RT-qPCR demonstrating mRNA expression levels of **(E)** KRT18. **(F)** METTL7A. **(G)** PRKCH.

To identify the key regulators in the TEX risk subgroup, first we verified the mRNA expression levels of these nine genes, and found that CCL20, CTLA4, GDF15, KRT18, and PERP were highly expressed in tumors, while KYNU, METTL7A, PRKCH, and BTG2 were highly expressed in normal tissues (Figure 10A). In addition, we used ROC diagnostic curves to screen for key regulators, and we found that the only ones with ROC>0.85 were KRT18, METTL7A, and PRKCH, and thus we concluded that these three genes were key regulatory genes for TEXRS (Figure 10B-D, Supplementary Figure 3A-F). We also plotted KM curves to verify the survival of these genes (Supplementary Figure 4A-I). These results showed that KRT18, METTL7A, and PRKCH were key regulatory genes for TEXRS. Finally, we assessed the expression of the three core genes in TEXRS in three cell lines, including one normal cell line (2B) and two lung adenocarcinoma cell lines (A549 and H1299) (Figure 10E-G). The results showed that METTL7A and PRKCH expression was significantly up-regulated in normal cell lines, while KRT18 expression was significantly up-regulated in tumor cell lines.

## Discussion

T cell exhaustion is defined as a state of dysfunction resulting from sustained exposure of T cells to antigenic and/or inflammatory signals in chronic infection or cancer 12. In this condition, exhausted T-cells, encompassing both effector and memory T cells, are unable to effectively eradicate infections and cancer 41. In this state, exhausted T-cells, including effector T cells and memory T cells, lose their ability to eliminate infection and cancer. However, studies have shown that inhibitory receptor overexpression is based on T-cell exhaustion. Blocking these receptors (e.g., PD-1 and CTLA-4) reverses the state and reactivates the immune response, thereby halting tumor progression 12, 41-44 which demonstrates the great potential of immune checkpoint blockade therapies in this regard. Unfortunately, despite the value of T-cell exhaustion in the development of many cancers, including LUAD, there have been few systematic studies of T-cell exhaustion in LUAD. Therefore, we developed a multi-biomarker model based on TEX-related genes, which can help physicians assess the prognosis and tumor microenvironment of LUAD patients and provide a theoretical basis for individualized precision therapy.

Using scRNA-seq data, Philip Bischoff et al identified genes associated with T-cell exhaustion from single-cell transcriptomes. The key modules most associated with T-cell exhaustion progression were then identified using the TCGA-LUAD data and the GSVA algorithm, and differential analysis of the TCGA-LUAD data was used to obtain differential genes. After analyzing the intersection of T cell exhaustion markers and differential genes, we identified 66 genes associated with T cell exhaustion in both single-cell and somatic transcriptomes. Subsequently, we created a new prognostic model using Lasso regression and one-way COX risk regression analysis which resulted in identifying 9 key genes. Significant prognostic differences between the two groups were observed, highlighting the independent prognostic worth of the TEX profile we established for LUAD. Our analyses of the ROC curve and calibration curve demonstrated that the TEX profile was a superior predictor of patient outcome. Additionally, we created a histogram that displayed the advantages of the TEX signature over other clinical indications in a promising manner.

Then, to gain more insight into the immunological characterization of TEXRS, we examined mutations in different TEXRS populations. As previously reported, missense variants were the most prevalent, followed by nonsense variants and shifted code deletions 45 TP53 mutations were more common in the high TEXRS group than in the low TEXPM group (56% vs. 42%), with the largest difference in mutation frequency between groups. TP53 mutations are not only commonly inherited in cancer, but also lead to aggressive malignancies and a poorer prognosis for patients 46, 47. TP53. Through the p53/TGF-b signaling pathway, TP53 can influence the cancer cell cycle. Finally, a better understanding of TME may help in the development of new therapies for LUAD or repair of TME to improve the effectiveness of immunotherapy. The composition of some immune cells differs between the two TEXRS groups; M0 and M2 macrophages are more common in the high TEXRS group, while cytotoxic CD8 T cells and CD4 T cells are more abundant in the low TEXRS group. Numerous studies have shown that dense infiltration of T cells, especially cytotoxic CD8 T cells, is a marker of good prognosis 48-50. In addition, based on the results of pathway enrichment, we found that the low TEXRS group had stronger immune pathways, while the high TEXRS group contained more immunosuppressive cells and oncogenic signals, as well as tumor- and metastasis-related signals, suggesting that the high TEXRS group exhibited immunosuppression and active tumor progression.

IPS data downloaded from TCIA can provide a predictive score for assessing a patient's response to immunotherapy 51, 52. The higher IPS in the low TEXRS group suggests that patients with low TEXRS may have a more favorable response to ICI therapy. This study suggests that TEXRS, which is not detected in LUAD, may be closely associated with immune infiltration in LUAD, suggesting a potential relevance of TEXRS in assessing immunotherapy responses. Surgical treatment, ablation or liver transplantation is an effective treatment for patients with early-stage LUAD and can significantly improve survival. Systematic therapy is the only option to improve survival in patients with advanced LUAD. In addition to immunotherapy-related drugs, we also tend to use some chemotherapeutic drugs, the vast majority of which improve survival time in LUAD patients in the low TEXRS group compared to the high TEXRS group.

In addition, we read the following article and found the AUCs of the prognostic models constructed by Wang Zi et al. by selecting the Arginine succinate gene were 0.68, 0.64 and 0.61 5; the AUCs of the prognostic models constructed by Chen Y et al. through the cuproptosis-related were 0.703, 0.663 and 0.574 53; the AUCs of the prognostic models constructed by Yang et al. The AUC values for the immune-related prognostic features were 0.718, 0.668, and 0.652 in Song C et al. 54. Our AUC results were 0.7, 0.7, and 0.69. These results suggest that TEXRS has good predictive ability to predict the prognosis of LUAD (Supplementary Figure 4A-C).

Based on these findings, we conclude that TEXRS is a good model for predicting survival time in LUAD patients and is closely related to the immune microenvironment. An in-depth study of TEXRS will facilitate the reversal of T-cell exhaustion and thus improve the efficacy of immunotherapy. Next, nine genomes comprise TEXRS: KYNU, CCL20, CTLA4, METTL7A, PRKCH, GDF15, BTG2, KRT18, and PERP. we screened by ROC curves that the key regulatory gene of TEXRS, KRT18 (Keratin 18), is thought to be overexpressed in most types of human tumor and correlated with clinical progression and prognosis 55-57. METTL7A is considered to be closely related to tumorigenesis, migration, drug resistance and prognosis of various tumors, and is also an early therapeutic target for lung adenocarcinoma 58-60. The protein kinase C (PKC) family promotes cell signaling and regulates the cell cycle; PRKCH (PKC-eta, PKCη) belongs to the PKC family and regulates cell proliferation, differentiation, and death 61-63. PRKCH has been shown to regulate cancer cell proliferation and increase chemotherapy resistance in a variety of cancers, including glioblastoma 64, breast cancer 65, and non-small cell lung cancer 66. Although the regulatory roles of these genes have been studied in various cancers, few researchers have systematically evaluated their prognostic value in LUAD. T-cell exhaustion has been less studied in lung adenocarcinoma, and thus we hope that the establishment of TEXRS will be used to improve the clinical management of lung adenocarcinoma patients.

Although the TEX signal we constructed is highly capable of identifying patients' immune status and predicting their prognosis, in our follow-up study, some limitations still need to be recognized and appropriate solutions found to address them. First, the TCGA-LUAD dataset we included is based on data from a public database, which can cause predictions to deviate from reality. While we have taken several approaches to try to avoid this, more data needs to be collected from LUAD patients to validate the utility of the model and the accuracy of immunotherapy predictions.

## Conclusion

As we first demonstrated, the TEX signature is a novel predictive biomarker and a potential therapeutic target for LUAD patients. In addition, the TEX signature can characterize the immune environment in LUAD patients and accurately evaluate the prognosis of LUAD patients, providing new ideas for clinical treatment of lung adenocarcinoma.

## Supplementary Material

Supplementary figures and tables.

## Figures and Tables

**Table 1:** TCGA-LUAD Clinical characteristics.

Characteristics	High Risk (N=250)	Low Risk (N=250)	Overall (N=500)	P-value
Age				
<=65	132 (52.8%)	115 (46.0%)	247 (49.4%)	0.315
>65	118 (47.2%)	135 (54.0%)	253 (50.6%)	
Gender				
Male	128 (51.2%)	102 (40.8%)	230 (46.0%)	0.025
Female	122 (51.2%)	148 (40.8%)	270 (54.0%)	
Stage				
I	106 (42.4%)	162 (64.8%)	268 (53.6%)	<0.001
II	70 (28.0%)	49 (19.6%)	119 (23.8%)	
III	55 (22.0%)	25 (10.0%)	80 (16.0%)	
IV	15 (6.0%)	10 (4.0%)	25 (5.0%)	
Unknown	4 (1.6%)	4 (1.6%)	8 (1.6%)	
T stage				
T1	61 (24.4%)	106 (42.4%)	167 (33.4%)	0.00608
T2	147 (58.8%)	120 (48.0%)	267 (53.4%)	
T3	28 (11.2%)	17 (6.8%)	45 (9.0%)	
T4	13 (5.2%)	5 (2.0%)	18 (3.6%)	
TX	1 (0.4%)	2 (0.8%)	3 (0.6%)	
N stage				
N0	140 (56.0%)	184 (73.6%)	324 (64.8%)	0.00175
N1	57 (22.8%)	37 (14.8%)	94 (18.8%)	
N2	49 (19.6%)	20 (8.0%)	69 (13.8%)	
N3	1 (0.4%)	1 (0.4%)	2 (0.4%)	
Unknown	3 (1.2%)	8 (3.2%)	11 (2.2%)	
M stage				
M0	173 (69.2%)	159 (63.6%)	332 (66.4%)	0.301
M1	15 (6.0%)	9 (3.6%)	24 (4.8%)	
Unknown	62 (24.8%)	82 (32.8%)	144 (28.8%)	

## References

[1] Siegel RL, Miller KD, Jemal A (2020). Cancer statistics, 2020. CA Cancer J Clin.

[2] Denisenko TV, Budkevich IN, Zhivotovsky B (2018). Cell death-based treatment of lung adenocarcinoma. Cell Death Dis.

[3] Imielinski M, Berger AH, Hammerman PS, Hernandez B, Pugh TJ, Hodis E (2012). Mapping the hallmarks of lung adenocarcinoma with massively parallel sequencing. Cell.

[4] Lahiri A, Maji A, Potdar PD, Singh N, Parikh P, Bisht B (2023). Lung cancer immunotherapy: progress, pitfalls, and promises. Mol Cancer.

[5] Wang Z, Zhang J, Shi S, Ma H, Wang D, Zuo C (2023). Predicting lung adenocarcinoma prognosis, immune escape, and pharmacomic profile from arginine and proline-related genes. Sci Rep.

[6] Song P, Li W, Guo L, Ying J, Gao S, He J (2022). Identification and Validation of a Novel Signature Based on NK Cell Marker Genes to Predict Prognosis and Immunotherapy Response in Lung Adenocarcinoma by Integrated Analysis of Single-Cell and Bulk RNA-Sequencing. Front Immunol.

[7] Sharma P, Hu-Lieskovan S, Wargo JA, Ribas A (2017). Primary, Adaptive, and Acquired Resistance to Cancer Immunotherapy. Cell.

[8] Zito Marino F, Bianco R, Accardo M, Ronchi A, Cozzolino I, Morgillo F (2019). Molecular heterogeneity in lung cancer: from mechanisms of origin to clinical implications. Int J Med Sci.

[9] Jin MZ, Jin WL (2020). The updated landscape of tumor microenvironment and drug repurposing. Signal Transduct Target Ther.

[10] McLane LM, Abdel-Hakeem MS, Wherry EJ (2019). CD8 T Cell Exhaustion During Chronic Viral Infection and Cancer. Annu Rev Immunol.

[11] Moskophidis D, Lechner F, Pircher H, Zinkernagel RM (1993). Virus persistence in acutely infected immunocompetent mice by exhaustion of antiviral cytotoxic effector T cells. Nature.

[12] Wherry EJ (2011). T cell exhaustion. Nat Immunol.

[13] Doering TA, Crawford A, Angelosanto JM, Paley MA, Ziegler CG, Wherry EJ (2012). Network analysis reveals centrally connected genes and pathways involved in CD8+ T cell exhaustion versus memory. Immunity.

[14] Day CL, Kaufmann DE, Kiepiela P, Brown JA, Moodley ES, Reddy S (2006). PD-1 expression on HIV-specific T cells is associated with T-cell exhaustion and disease progression. Nature.

[15] Wherry EJ, Kurachi M (2015). Molecular and cellular insights into T cell exhaustion. Nat Rev Immunol.

[16] Zhang Z, Chen L, Chen H, Zhao J, Li K, Sun J (2022). Pan-cancer landscape of T-cell exhaustion heterogeneity within the tumor microenvironment revealed a progressive roadmap of hierarchical dysfunction associated with prognosis and therapeutic efficacy. EBioMedicine.

[17] Bischoff P, Trinks A, Obermayer B, Pett JP, Wiederspahn J, Uhlitz F (2021). Single-cell RNA sequencing reveals distinct tumor microenvironmental patterns in lung adenocarcinoma. Oncogene.

[18] Johnson DB, Estrada MV, Salgado R, Sanchez V, Doxie DB, Opalenik SR (2016). Melanoma-specific MHC-II expression represents a tumour-autonomous phenotype and predicts response to anti-PD-1/PD-L1 therapy. Nat Commun.

[19] Wei SC, Duffy CR, Allison JP (2018). Fundamental Mechanisms of Immune Checkpoint Blockade Therapy. Cancer Discov.

[20] Langfelder P, Horvath S (2008). WGCNA: an R package for weighted correlation network analysis. BMC Bioinformatics.

[21] Hanzelmann S, Castelo R, Guinney J (2013). GSVA: gene set variation analysis for microarray and RNA-seq data. BMC Bioinformatics.

[22] Wu T, Hu E, Xu S, Chen M, Guo P, Dai Z (2021). clusterProfiler 4.0: A universal enrichment tool for interpreting omics data. Innovation (Camb).

[23] Mroz EA, Rocco JW (2013). MATH, a novel measure of intratumor genetic heterogeneity, is high in poor-outcome classes of head and neck squamous cell carcinoma. Oral Oncol.

[24] Rajput A, Bocklage T, Greenbaum A, Lee JH, Ness SA (2017). Mutant-Allele Tumor Heterogeneity Scores Correlate With Risk of Metastases in Colon Cancer. Clin Colorectal Cancer.

[25] Ma D, Jiang YZ, Liu XY, Liu YR, Shao ZM (2017). Clinical and molecular relevance of mutant-allele tumor heterogeneity in breast cancer. Breast Cancer Res Treat.

[26] Mroz EA, Tward AD, Hammon RJ, Ren Y, Rocco JW (2015). Intra-tumor genetic heterogeneity and mortality in head and neck cancer: analysis of data from the Cancer Genome Atlas. PLoS Med.

[27] Newman AM, Liu CL, Green MR, Gentles AJ, Feng W, Xu Y (2015). Robust enumeration of cell subsets from tissue expression profiles. Nat Methods.

[28] Yoshihara K, Shahmoradgoli M, Martínez E, Vegesna R, Kim H, Torres-Garcia W (2013). Inferring tumour purity and stromal and immune cell admixture from expression data. Nat Commun.

[29] Jiang P, Gu S, Pan D, Fu J, Sahu A, Hu X (2018). Signatures of T cell dysfunction and exclusion predict cancer immunotherapy response. Nat Med.

[30] Zeng D, Li M, Zhou R, Zhang J, Sun H, Shi M (2019). Tumor Microenvironment Characterization in Gastric Cancer Identifies Prognostic and Immunotherapeutically Relevant Gene Signatures. Cancer Immunol Res.

[31] Ulloa-Montoya F, Louahed J, Dizier B, Gruselle O, Spiessens B, Lehmann FF (2013). Predictive gene signature in MAGE-A3 antigen-specific cancer immunotherapy. J Clin Oncol.

[32] Lauss M, Donia M, Harbst K, Andersen R, Mitra S, Rosengren F (2017). Mutational and putative neoantigen load predict clinical benefit of adoptive T cell therapy in melanoma. Nat Commun.

[33] Zeng D, Ye Z, Wu J, Zhou R, Fan X, Wang G (2020). Macrophage correlates with immunophenotype and predicts anti-PD-L1 response of urothelial cancer. Theranostics.

[34] Vogelstein B, Papadopoulos N, Velculescu VE, Zhou S, Diaz LA Jr, Kinzler KW (2013). Cancer genome landscapes. Science.

[35] Dagogo-Jack I, Shaw AT (2018). Tumour heterogeneity and resistance to cancer therapies. Nat Rev Clin Oncol.

[36] Xia Z, Qing B, Wang W, Gu L, Chen H, Yuan Y (2021). Formation, contents, functions of exosomes and their potential in lung cancer diagnostics and therapeutics. Thorac Cancer.

[37] Xu-Monette ZY, Zhou J, Young KH (2018). PD-1 expression and clinical PD-1 blockade in B-cell lymphomas. Blood.

[38] Qin S, Xu L, Yi M, Yu S, Wu K, Luo S (2019). Novel immune checkpoint targets: moving beyond PD-1 and CTLA-4. Mol Cancer.

[39] Chauvin JM, Zarour HM (2020). TIGIT in cancer immunotherapy. J Immunother Cancer.

[40] Yang K, Halima A, Chan TA (2023). Antigen presentation in cancer - mechanisms and clinical implications for immunotherapy. Nat Rev Clin Oncol.

[41] Nguyen LT, Ohashi PS (2015). Clinical blockade of PD1 and LAG3-potential mechanisms of action. Nat Rev Immunol.

[42] Pauken KE, Wherry EJ (2015). Overcoming T cell exhaustion in infection and cancer. Trends Immunol.

[43] Barber DL, Wherry EJ, Masopust D, Zhu B, Allison JP, Sharpe AH (2006). Restoring function in exhausted CD8 T cells during chronic viral infection. Nature.

[44] Schietinger A, Greenberg PD (2014). Tolerance and exhaustion: defining mechanisms of T cell dysfunction. Trends Immunol.

[45] Perez Sayans M, Chamorro Petronacci CM, Lorenzo Pouso AI, Padin Iruegas E, Blanco Carrion A, Suarez Penaranda JM (2019). Comprehensive Genomic Review of TCGA Head and Neck Squamous Cell Carcinomas (HNSCC). J Clin Med.

[46] Olivier M, Langerod A, Carrieri P, Bergh J, Klaar S, Eyfjord J (2006). The clinical value of somatic TP53 gene mutations in 1,794 patients with breast cancer. Clin Cancer Res.

[47] Vousden KH, Prives C (2005). P53 and prognosis: new insights and further complexity. Cell.

[48] Fridman WH, Zitvogel L, Sautes-Fridman C, Kroemer G (2017). The immune contexture in cancer prognosis and treatment. Nat Rev Clin Oncol.

[49] Gentles AJ, Newman AM, Liu CL, Bratman SV, Feng W, Kim D (2015). The prognostic landscape of genes and infiltrating immune cells across human cancers. Nat Med.

[50] Bindea G, Mlecnik B, Tosolini M, Kirilovsky A, Waldner M, Obenauf AC (2013). Spatiotemporal dynamics of intratumoral immune cells reveal the immune landscape in human cancer. Immunity.

[51] Chumsri S, Sokol ES, Soyano-Muller AE, Parrondo RD, Reynolds GA, Nassar A (2020). Durable Complete Response With Immune Checkpoint Inhibitor in Breast Cancer With High Tumor Mutational Burden and APOBEC Signature. J Natl Compr Canc Netw.

[52] Van Allen EM, Miao D, Schilling B, Shukla SA, Blank C, Zimmer L (2015). Genomic correlates of response to CTLA-4 blockade in metastatic melanoma. Science.

[53] Chen Y, Tang L, Huang W, Zhang Y, Abisola FH, Li L (2022). Identification and validation of a novel cuproptosis-related signature as a prognostic model for lung adenocarcinoma. Front Endocrinol (Lausanne).

[54] Song C, Guo Z, Yu D, Wang Y, Wang Q, Dong Z (2020). A Prognostic Nomogram Combining Immune-Related Gene Signature and Clinical Factors Predicts Survival in Patients With Lung Adenocarcinoma. Front Oncol.

[55] Lai YC, Cheng CC, Lai YS, Liu YH (2017). Cytokeratin 18-associated Histone 3 Modulation in Hepatocellular Carcinoma: A Mini Review. Cancer Genomics Proteomics.

[56] Fortier AM, Asselin E, Cadrin M (2013). Keratin 8 and 18 loss in epithelial cancer cells increases collective cell migration and cisplatin sensitivity through claudin1 up-regulation. J Biol Chem.

[57] Zhang J, Hu S, Li Y (2019). KRT18 is correlated with the malignant status and acts as an oncogene in colorectal cancer. Biosci Rep.

[58] Zehmer JK, Bartz R, Bisel B, Liu P, Seemann J, Anderson RG (2009). Targeting sequences of UBXD8 and AAM-B reveal that the ER has a direct role in the emergence and regression of lipid droplets. J Cell Sci.

[59] Zehmer JK, Bartz R, Liu P, Anderson RG (2008). Identification of a novel N-terminal hydrophobic sequence that targets proteins to lipid droplets. J Cell Sci.

[60] Guo T, Ma H, Zhou Y (2019). Bioinformatics analysis of microarray data to identify the candidate biomarkers of lung adenocarcinoma. PeerJ.

[61] Porter SN, Magee JA (2017). PRKCH regulates hematopoietic stem cell function and predicts poor prognosis in acute myeloid leukemia. Exp Hematol.

[62] Basu A (2019). The Enigmatic Protein Kinase C-eta. Cancers (Basel).

[63] Pal D, Outram SP, Basu A (2012). Novel regulation of protein kinase C-eta. Biochem Biophys Res Commun.

[64] Uht RM, Amos S, Martin PM, Riggan AE, Hussaini IM (2007). The protein kinase C-eta isoform induces proliferation in glioblastoma cell lines through an ERK/Elk-1 pathway. Oncogene.

[65] Masso-Welch PA, Winston JS, Edge S, Darcy KM, Asch H, Vaughan MM (2001). Altered expression and localization of PKC eta in human breast tumors. Breast Cancer Res Treat.

[66] Krasnitsky E, Baumfeld Y, Freedman J, Sion-Vardy N, Ariad S, Novack V (2012). PKCeta is a novel prognostic marker in non-small cell lung cancer. Anticancer Res.

