# Supplementary material for: Identification of T-cell exhaustion-related genes and prediction of their immunotherapeutic role in lung adenocarcinoma
Source: J Cancer. 2024 Feb 25;15(8):2160–78. doi: 10.7150/jca.92839 (PMC10937285; doi:10.7150/jca.92839)
Supplement: Supplementary file 1 — Supplementary figures and tables. [file jcav15p2160s1.zip › Supplementary.docx]

**Supplementary Figures:**

**
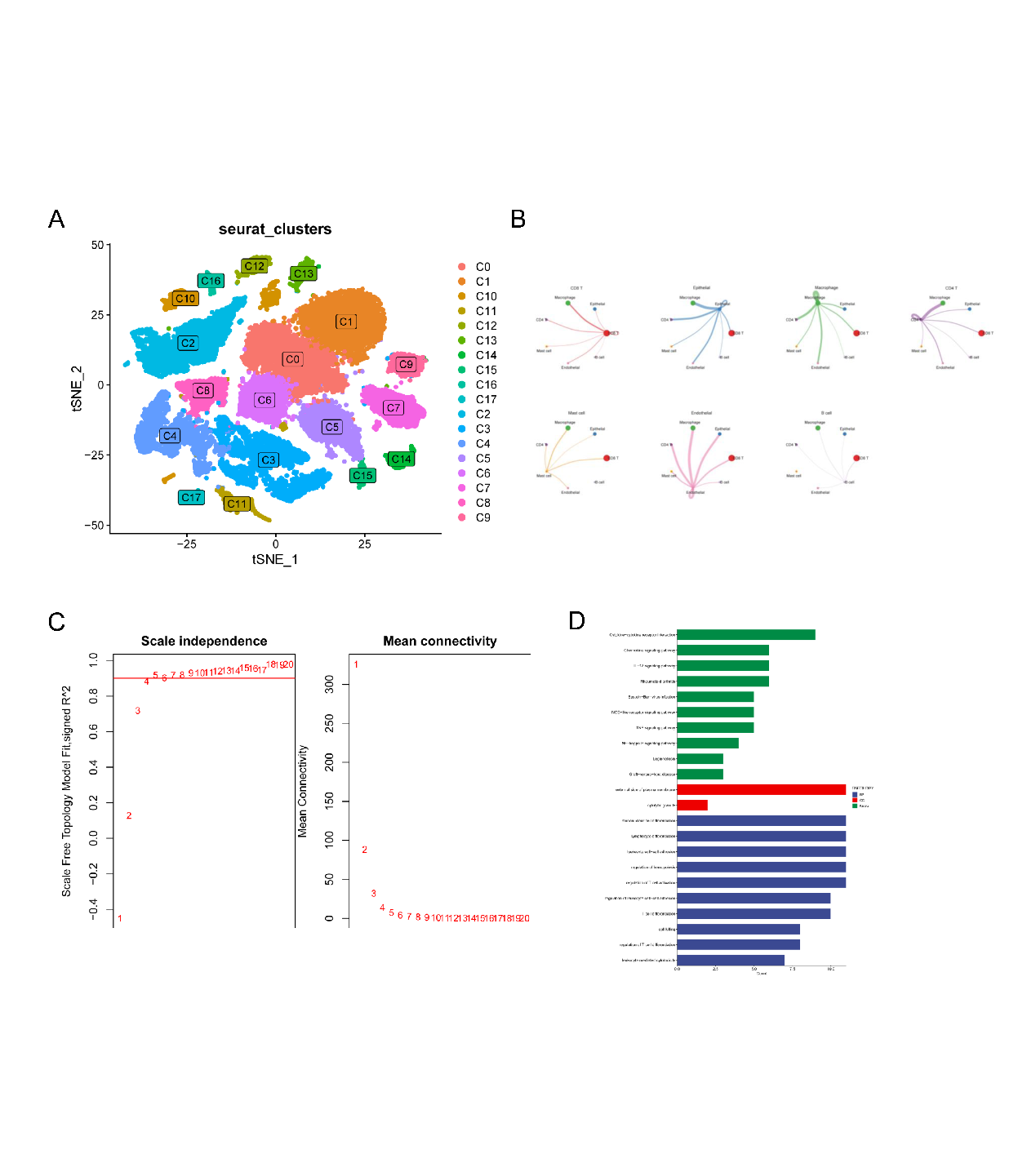
**

**Supplementary Figure.1 Single-cell and WGCNA identification of genes and gene enrichment analysis.**

(A)TSNE demonstrates cell clusters. (B)intercellular communication. (C)WGCNA Screening Soft Queering. (D)Gene enrichment pathway.


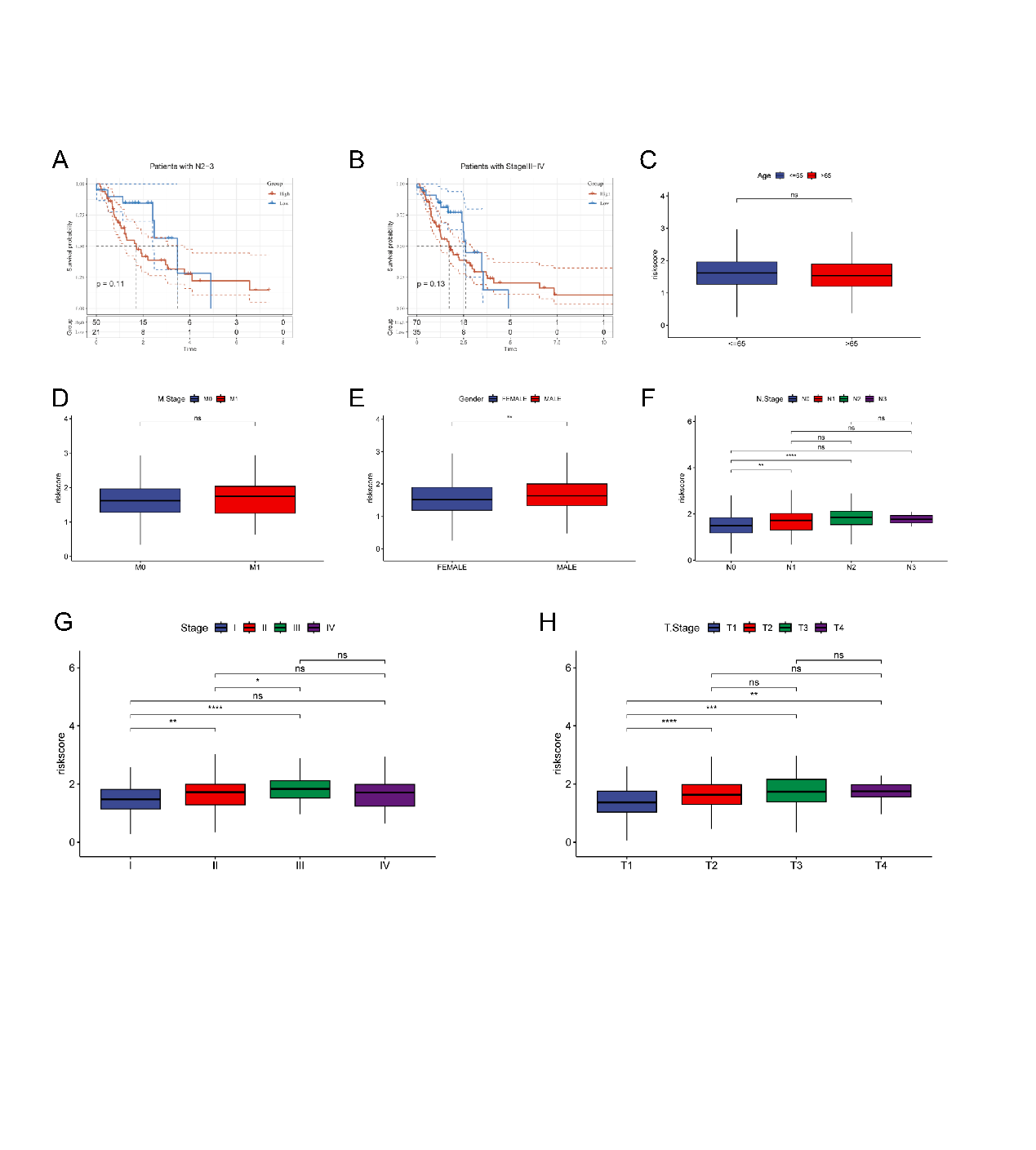


**Supplementary Figure.2 Clinical features of TEXRS.**

(A)km curves for patients N2 and N3.(B) km curves for staging III and IV patients. Box plot showing different groups, (C)Age, (D)M.Stage, (E)Gender, (F)N.Stage, (G)Stage, (H)T.Stage.


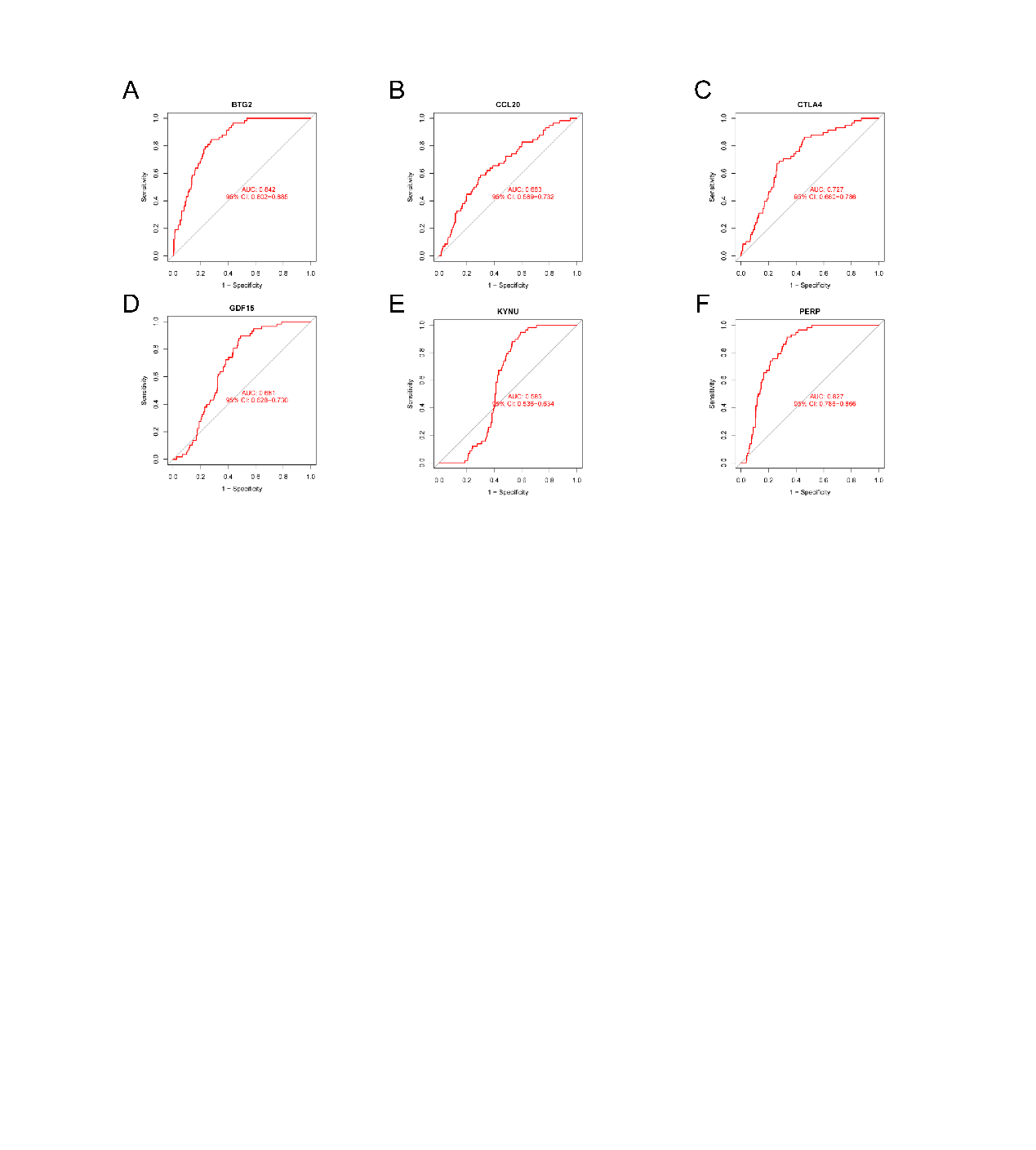


**Supplementary Figure.3 ROC diagnostic curve.**

ROC diagnostic curve for the TEXRS gene including BTG2(A),CCL20(B),CTLA4(C),GDF15(D),KYNU(E),PERP(F).


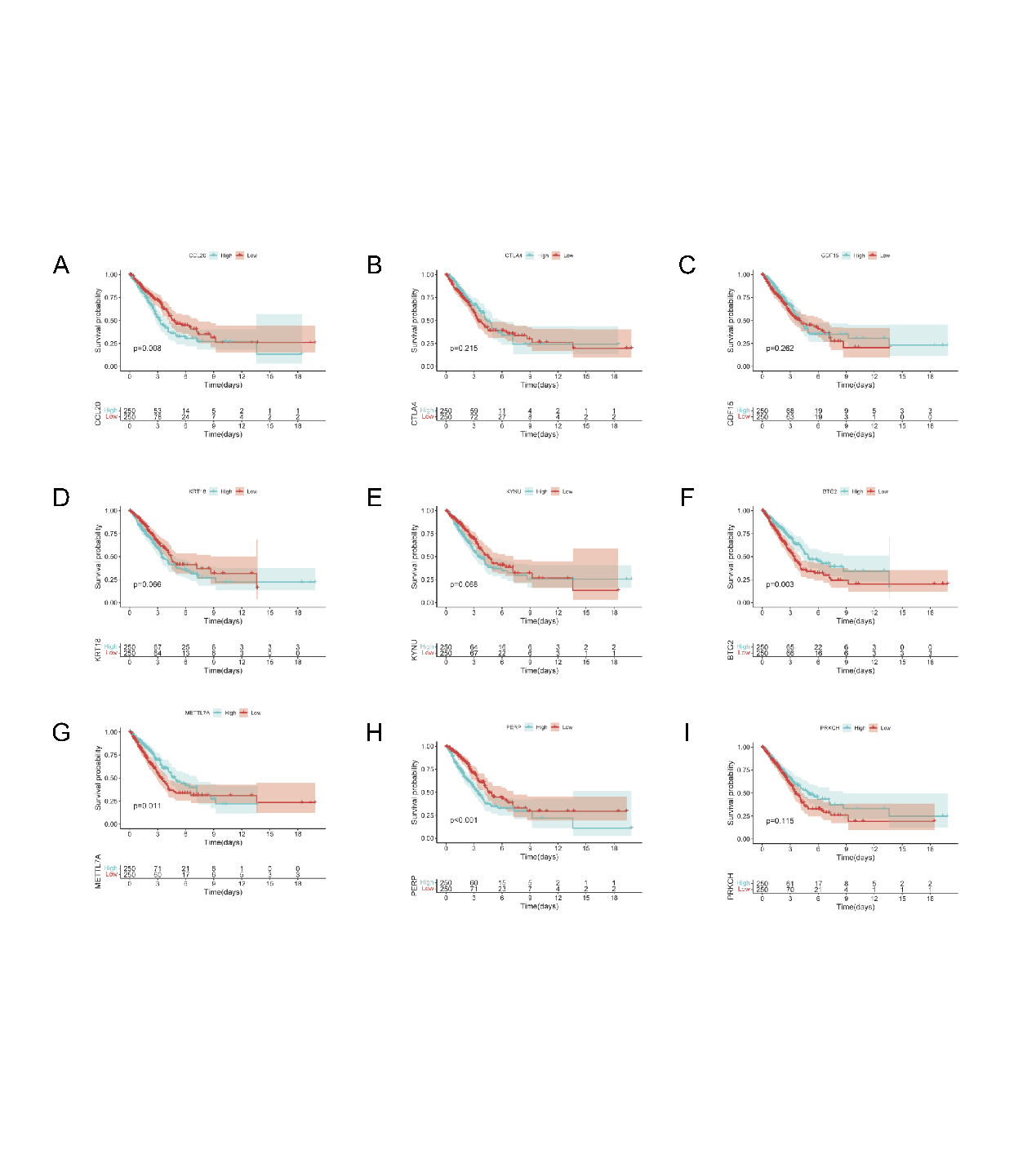


**Supplementary Figure.4 KM survival curves for the TEXRS gene.**

KM survival curves for the TEXRS gene including CCL20(A),CTLA4(B),GDF15(C),KRT18(D),KYNU(E),BTG2(F),METTL7A(G),PERP(H),PRKCH(I).


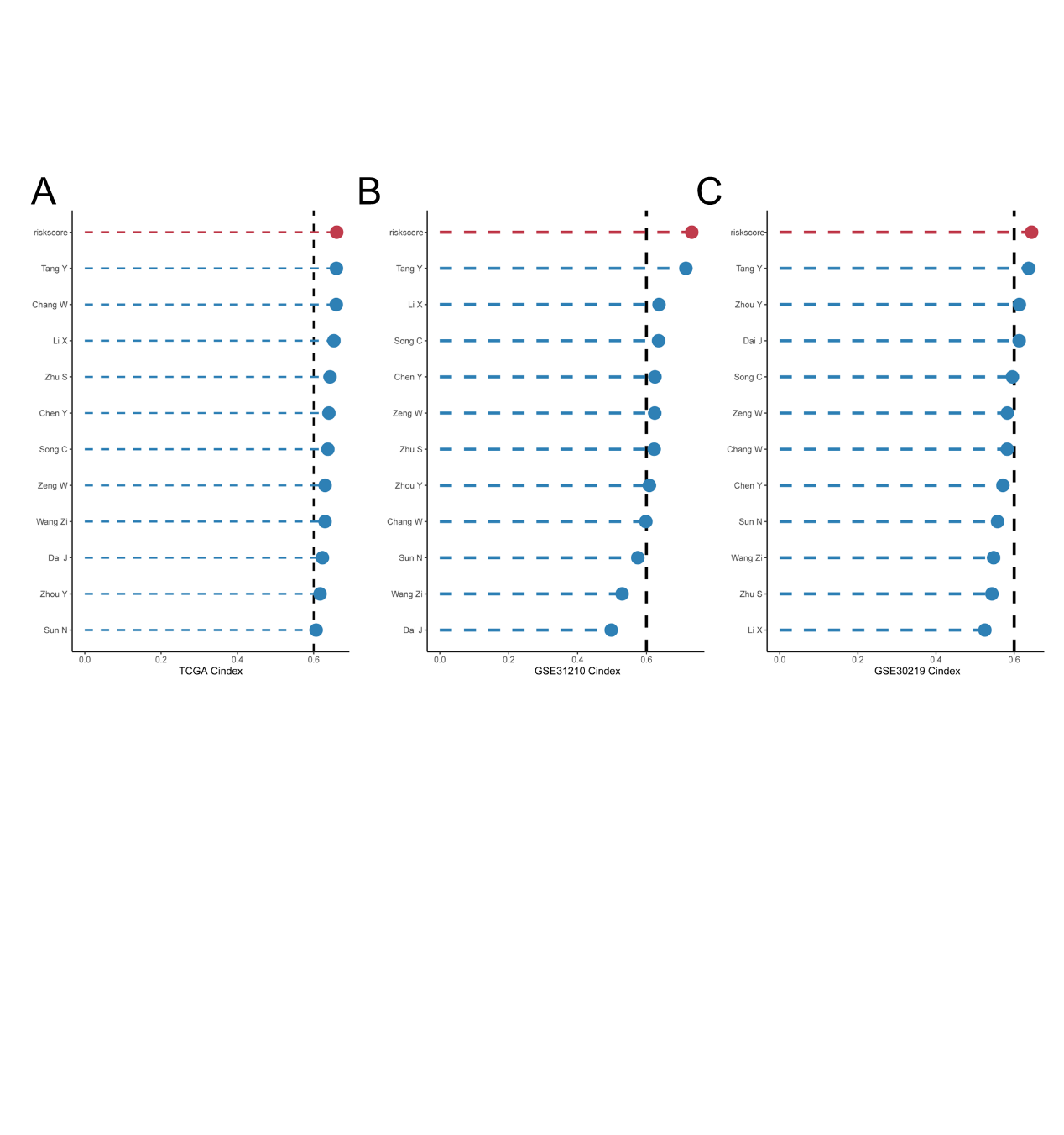


**Supplementary Figure.5 Comparison of models.**

Comparison with the C-index in other published articles, The results show that riskscore has good predictive power.(A) Comparison in TCGA-LUAD. (B) Comparison in GSE31210. (C) Comparison in GSE30219.
